# Artificial Intelligence-Assisted Conductive Hydrogel Dressings for Refractory Wounds Monitoring

**DOI:** 10.1007/s40820-025-01834-w

**Published:** 2025-07-03

**Authors:** Yumo She, He Liu, Hailiang Yuan, Yiqi Li, Xunjie Liu, Ruonan Liu, Mengyao Wang, Tingting Wang, Lina Wang, Meihan Liu, Wenyu Wan, Ye Tian, Kai Zhang

**Affiliations:** 1 Department of Gastroenterology, Endoscopic Center, Shengjing Hospital of China Medical University, Shenyang, 110004 People’s Republic of China; 2 College of Medicine and Biological Information Engineering, Northeastern University, Shenyang, 110169 People’s Republic of China; 3 Department of Gastroenterology, First Affiliated Hospital of Jinzhou Medical University, Jinzhou, 121000 People’s Republic of China; 4 Department of Dermatology, The First Hospital of China Medical University, Shenyang, 110001 People’s Republic of China; 5 Key Laboratory of Immunodermatology, Ministry of Education, and National Health Commission, National Joint Engineering Research Center for Theranostics of Immunological Skin Diseases, Shenyang, 110006 People’s Republic of China; 6 Foshan Graduate School of Innovation, Northeastern University, Foshan, 528300 People’s Republic of China; 7 Engineering Research Center of Ministry of Education for Minimally Invasive Gastrointestinal Endoscopic Techniques, Shengjing Hospital of China Medical University, Shenyang, 110004 People’s Republic of China

**Keywords:** Artificial intelligence, Conductive hydrogels, Refractory wounds, Wound healing, Wound monitoring

## Abstract

Refractory wounds cause significant harm to the health of patients and the most common treatments in clinical practice are surgical debridement and wound dressings. However, certain challenges, including surgical difficulty, lengthy recovery times, and a high recurrence rate persist. Conductive hydrogel dressings with combined monitoring and therapeutic properties have strong advantages in promoting wound healing due to the stimulation of endogenous current on wounds and are the focus of recent advancements. Therefore, this review introduces the mechanism of conductive hydrogel used for wound monitoring and healing, the materials selection of conductive hydrogel dressings used for wound monitoring, focuses on the conductive hydrogel sensor to monitor the output categories of wound status signals, proving invaluable for non-invasive, real-time evaluation of wound condition to encourage wound healing. Notably, the research of artificial intelligence (AI) model based on sensor derived data to predict the wound healing state, AI makes use of this abundant data set to forecast and optimize the trajectory of tissue regeneration and assess the stage of wound healing. Finally, refractory wounds including pressure ulcers, diabetes ulcers and articular wounds, and the corresponding wound monitoring and healing process are discussed in detail. This manuscript supports the growth of clinically linked disciplines and offers motivation to researchers working in the multidisciplinary field of conductive hydrogel dressings.

## Highlights

- The advantages and selection of conductive materials, including conductive polymers and inorganic nanoparticles are discussed in detail.
- Signal output categories of the conductive hydrogel dressing to monitor wound conditions, notably, AI-based wound monitoring and prediction is highlighted.
- The review analyzes the application, current challenges and potential prospects of conductive hydrogel dressings for the different refractory wounds monitoring.

## Graphical abstract

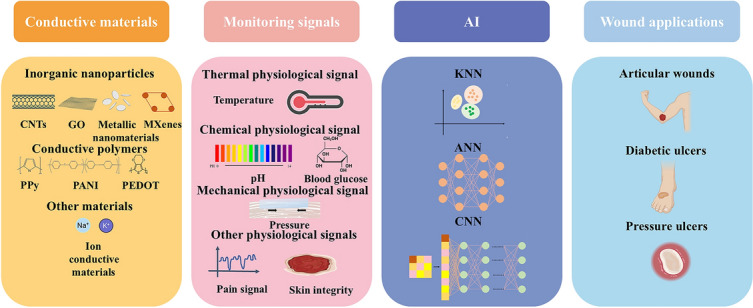

## Introduction

Refractory wounds refer to wounds with multiple and complex injury factors, slow healing, and no obvious tendency to heal after treatment for 4 weeks [1]. Pressure ulcers, diabetes ulcers, articular wounds, burns and ischemic ulcers are typical manifestations of chronic refractory diseases. Age-related articular tissue degradation, systemic disease effects (diabetic hyperglycemia toxicity, long-term nutritional deficiency in bed), inadequate infection control (multidrug-resistant bacteria and biofilm formation), treatment methods and management flaws (poor dressing selection or patient compliance), and local microenvironment imbalance (ischemia, hypoxia and chronic inflammation) are some of the factors that contribute to the difficulty of healing refractory wounds [2]. These conditions disrupt the normal wound-healing process, leading to delayed healing and various complications including infections. Moreover, they heighten the risk of multiple diseases stemming from large-vessel and microvascular injuries. The adverse effects of these diseases are not confined to the wound site, they can also have a detrimental impact on multiple vital organs, such as the heart, brain, kidneys, eyes, and more [3, 4]. Patients have experienced severe outcomes from this kind of wound, which has presented a significant financial load and difficulty for the hospital system as well as society [5]. Conventional wound dressings, for instance, gauze, cotton, and hemostatic sponge, may stick to the site tightly while being used, increasing discomfort and bleeding and perhaps causing secondary wound damage. In recent years, hydrogels have become more popular as wound dressings. Their exceptional hydrophilicity, biocompatibility, and resemblance to the extracellular matrix (ECM) allow them to support and encourage tissue regeneration, cell migration, and proliferation in addition to efficiently absorbing wound exudates [6]. However, most of them are unable to monitor the condition of the wound in real time, and treating the wound blindly may lessen the treatment effect [7]. To assess healing status in real time, get early warning of risk events such as infection and ischemia, and realize treatment plan optimization, wound monitoring is essential in the medical field. Its primary value is the dynamic collection of wound area, depth, microenvironment parameters (including pH, temperature, and inflammatory factors), and systemic indicators. It is essential for enhancing the prognosis and standard of care for patients with refractory wounds [8].

Conductive hydrogels exhibit exceptional suitability for wound healing and monitoring. Conductive hydrogels are three-dimensional materials with a high-water content that exhibit conductive qualities. Through a variety of techniques, conductive polymers, inorganic nanoparticles, or ions conductive materials are mixed into the hydrogel system to create them. Conductive hydrogels present a distinct advantage in wound healing. Their electroactive components can stimulate the generation of endogenous current, decrease swelling around wounds, direct keratinocyte migration at the wound site, improve re-epithelialization, promote angiogenesis, control several genes involved in wound healing and have antibacterial properties, playing a key role in the wound healing process. Using dressings to respond to changes in the wound microenvironment (including temperature, pH, and blood glucose) ensures quick and accurate treatment, meeting the treatment needs of every phase in the wound healing process. Since the wound healing process is divided into several stages, each stage has its unique microenvironmental characteristics. Conductive hydrogels can use integrated sensors or smart materials to transform the shifting signals in the microenvironment into electrical impulses. Concurrently, conductive hydrogels with wound monitoring capabilities can promptly address the “black box” [9] period of wound healing—a blind period that is unable to be observed and monitored to provide on-demand care and enhance the healing outcome. Thus, conductive hydrogel dressings with wound monitoring and treatment capabilities have emerged as a research hotspot in the field of wound dressings, by assessing the wound healing microenvironment and treating wounds as necessary, they offer a novel approach to monitoring and treating chronic refractory wounds. And research on the creation of intelligent wound dressings has also experienced notable growth [10].

AI technology is a viable scientific approach to enhance the effect of conductive hydrogel dressings on the treatment of chronic refractory wounds. Conductive hydrogel is based on the ion/electron conduction characteristics of conductive materials, integrated temperature, pH, pressure, glucose and other sensor modules continuously monitor wound microenvironment parameters (including local temperature rise and abnormal pressure fluctuations in the early stages of infection). Through the hydrogel’s conductive network, the physiological signals are transformed into electrical impulses, which are then instantly transmitted to the data endpoint via the wireless transmission module. To create a thorough evaluation system, an AI model combines data from multiple sources that are gathered by conductive hydrogel to conduct further analysis and exploration [11]. In the past five years, there has been a growing interest in the integrated monitoring and treatment capabilities of conductive hydrogel sensors combined with human-computer interfaces. By gathering physiological signals from the human body, smart conductive sensors integrated into wound dressings can offer real-time, non-invasive assessment of wound characteristics as well as alternate methods for disease diagnosis and health monitoring [12]. Through continuous and remote monitoring made possible by this integration, medical professionals can gather firsthand data on the dynamic progression of refractory wounds, helping to prevent infections, shorten treatment durations, and lower treatment expenses [13]. Through AI-powered diagnostic platforms integrating deep learning algorithms, clinicians can implement real-time wound status tracking and adaptive therapeutic management, enabling predictive analytics for healing trajectory assessment. This intelligent system enables precisely timed intervention strategies. These strategies not only reduce the risks of the transition from acute to chronic pathology but also prevent the deterioration of refractory wounds by continuously optimizing the microenvironment. Owing to the advancement of this technology, advanced wound monitoring technology has emerged and evolved. The combination of AI prediction has significantly improved the capability of conductive hydrogel dressings in wound monitoring [14]. AI algorithms can identify intricate patterns and correlations in monitoring parameters when trained on various datasets. This makes it possible to detect abnormal wounds at an early stage, allowing for timely intervention and the formulation of personalized treatment plans tailored to the specific circumstances of each patient’s wound [15]. There are now many opportunities for the real-time gathering and transmission of emergency signal data for wound healing based on the use of AI technology.

**Fig. 1:**
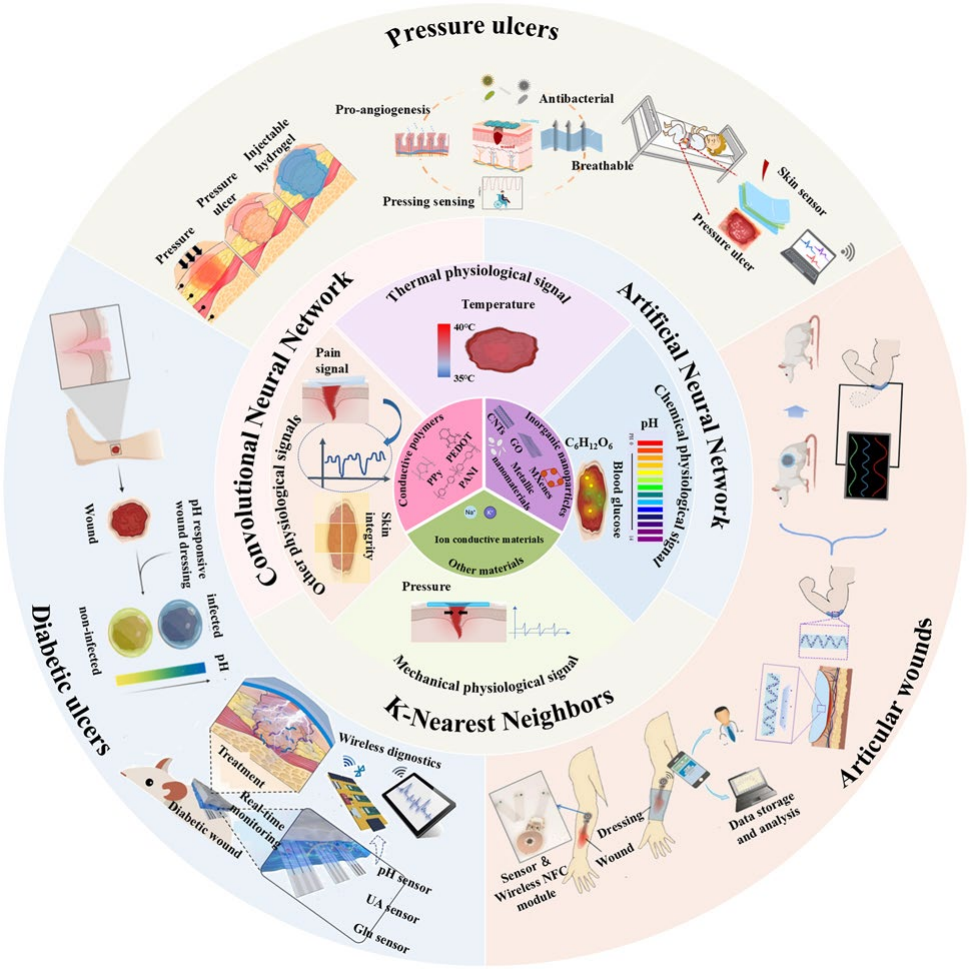
Common materials, monitoring mechanisms and applications of conductive hydrogel dressings. Pressure ulcers: Reproduced with permission from Refs. [16–18]. Copyright 2022, Elsevier. And Copyright 2025, Elsevier. And Copyright 2022, Elsevier. Articular wounds: Reproduced with permission from Refs. [19, 20]. Copyright 2023, American Chemical Society. And Copyright 2024, Elsevier. Diabetic ulcers: Reproduced with permission from Refs. [21, 22]. Copyright 2023, Elsevier. And Copyright 2023, Elsevier

**Fig. 2:**
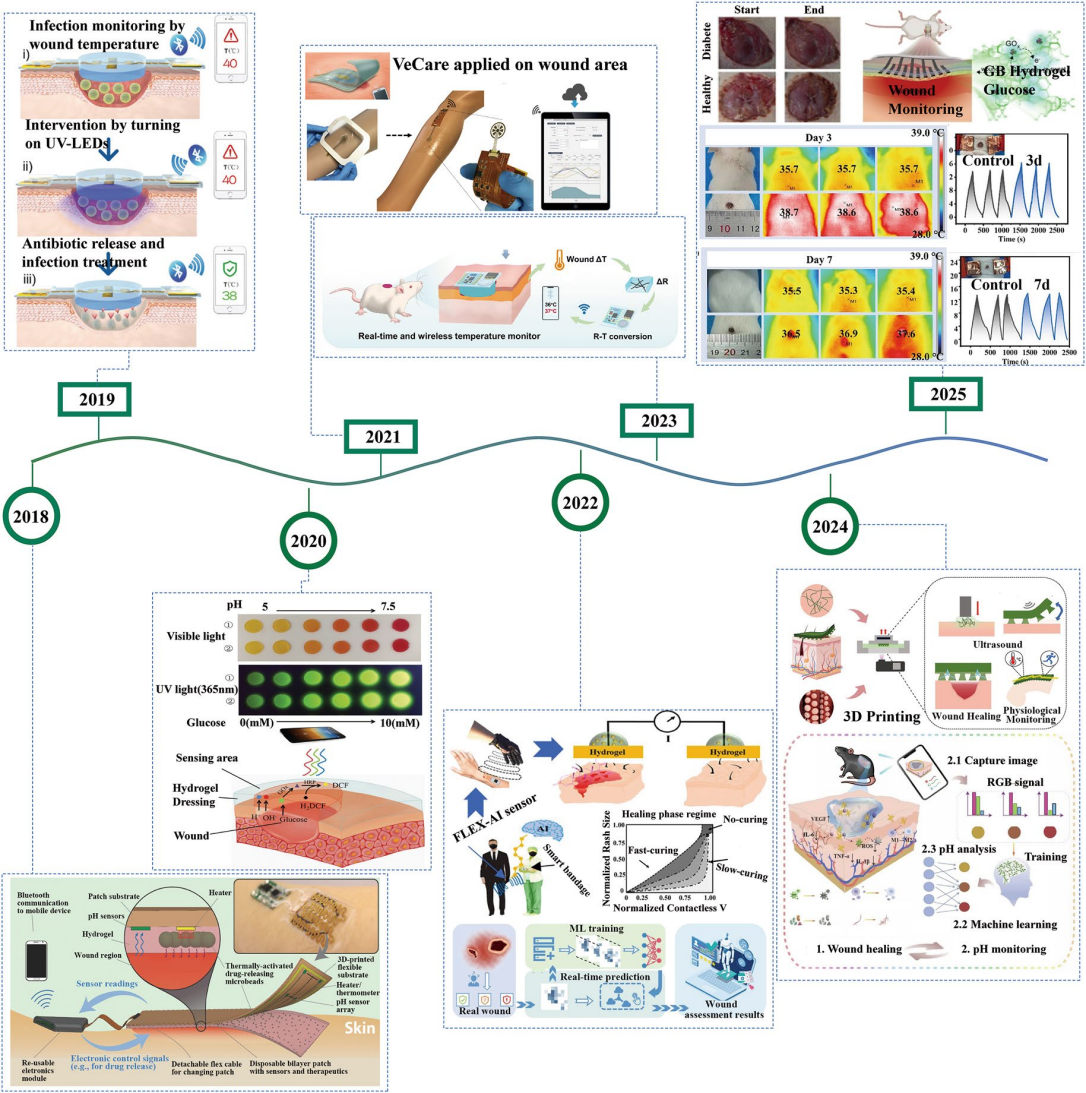
Chronological development of conductive hydrogel dressings. 2018: The smart bandage communicates wirelessly with computers and smartphones to monitor wound pH and regulate the temperature to release antibiotics locally as needed. Reproduced with permission from Ref. [23]. Copyright 2018, Wiley–VCH. 2019: The temperature sensor integrated with the conductive hydrogel dressing collects the wound temperature, transmits it to the smartphone in real time through Bluetooth communication, and uses UV-LED to remotely control the in situ release of antibiotics. Reproduced with permission from Ref. [9]. Copyright 2020, Wiley–VCH. 2020: This conductive hydrogel dressing can simultaneously monitor the pH value and glucose level in diabetic wounds by colorimetry, and be collected by smartphones and converted into RGB signals to quantify wound parameters. Reproduced with permission from Ref. [24]. Copyright 2020, Wiley–VCH. 2021: The platform integrates a sensor array for measuring inflammatory mediators, microbial load, and physicochemical parameters for wireless data readout based on smartphones. Reproduced with permission from Ref. [25]. Copyright 2021, American Association for the Advancement of Science. 2022: Upper: Monitoring chronic skin wound healing based on contact pH-responsive voltage output combined with deep ANN machine learning. Lower: Conductive hydrogel dressing realizes wound pH value monitoring through colorimetry and personalized wound management based on CNN algorithm. Reproduced with permission from Refs. [26, 27]. Copyright 2022, Elsevier. And Copyright 2022, American Chemical Society. 2023: Resistance conductive hydrogel dressing is integrated with a wireless Bluetooth module for real-time wound temperature monitoring. Reproduced with permission from Ref. [28]. Copyright 2023, Royal Society of Chemistry. 2024: Upper: US mediated and 3D printed conductive hydrogel dressings realize wound temperature monitoring. Lower: Conductive hydrogel with the characteristics of visual pH monitoring is integrated with smartphones to achieve image visualization and use KNN algorithm to further achieve wound pH assessment. Reproduced with permission from Refs. [29, 30]. Copyright 2024, Elsevier. And Copyright 2024, Elsevier. 2025: Upper: Simultaneous monitoring of glucose in wound exudate and sweat glucose through photoelectric dual signals. Lower: Conductive hydrogel dressings monitor the temperature change at the wound with resistance change and infrared image. Reproduced with permission from Refs. [31, 32]. Copyright 2025, Elsevier. And Copyright 2025, Wiley–VCH

In this manuscript, we introduce the mechanism of conductive hydrogel dressings used for wound monitoring and healing, the materials selection of conductive hydrogel dressings, focus on the signal output categories of conductive hydrogel dressings used to monitor wound conditions in detail, and the related research of AI technology combination based on sensor derived data to predict the state of wound healing. Moreover, we discussed wound monitoring and healing process corresponding to chronic refractory wounds involving pressure ulcers, diabetes ulcers, and articular wounds (Fig. 1). Finally, by covering these key aspects, we highlight the potential progress and research opportunities of the integrated conductive hydrogel wound monitoring and treatment system in the advanced wound monitoring and personalized medicine field, providing more enlightenment and guidance for researchers and medical workers in related fields (Fig. 2).

## Conductive Hydrogel Dressings for Refractory Wounds Healing and Monitoring

Electroactive conductive hydrogels are thought to be crucial instruments for accelerating refractory wound healing. The intricate process of wound healing and restoration involves hemostasis, inflammation, proliferation, and remodeling phases [33]. Transepithelial potential (TEP) is the voltage differential between the two sides of epithelial cells in healthy tissues within an organism [34, 35]. Endogenous currents are produced at the wound site due to the passage of electric charges when a wound forms, and they can be stimulated to successfully promote and expedite wound closure [36]. On one hand, the high permeability of conductive hydrogels creates a moist wound environment—ideal for healing by delivering adequate oxygen and nutrients [37], on the other hand, their electroactive materials generate an electric current that modulates the wound’s TEP, sealing the wound and regulating the activity of electrically responsive cells, such as keratinocytes and fibroblasts. These cells are essential for wound healing since they migrate more forcefully and can induce the production of endogenous currents [38–40]. Additionally, the wound may produce an excessive amount of reactive oxygen species (ROS), which raises the risk of infection, slows tissue regeneration, and results in poor wound healing. Conductive hydrogels with electrically active materials such as conductive polymers and inorganic nanomaterials exhibit remarkable antioxidant capacity and highly efficient free radical scavenging ability. By eliminating too much ROS, conductive hydrogel dressings containing electroactive elements can help encourage tissue regeneration following oxidative injury [41–43]. Thus, conductive hydrogel dressings containing electroactive elements can help encourage tissue regeneration following oxidative injury by eliminating excessive ROS [44]. Therefore, conductive hydrogel offers a wide range of potential applications in the wound repair field as a functional wound dressing.

In addition, conductive hydrogel dressing is a valuable tool for tracking refractory wound healing. Wound monitoring is especially important for refractory wounds since the healing state of the wound must be taken into account while adjusting subsequent treatment. The suboptimal diagnostic precision and delayed therapeutic feedback in wound management stem from persistent dependence on conventional monitoring paradigms that combine subjective visual evaluation with intermittent hospital-based laboratory analyses. While existing electrochemical biosensing platforms demonstrate partial capability for wound assessment, their single-parameter detection limitations preclude the simultaneous acquisition of multimodal diagnostic data required for comprehensive pathophysiological evaluation [45, 46]. According to some research, the current conductive hydrogel can be employed as a sensor for real-time wound healing monitoring as well as a wound dressing. By tracking physiological signals including pH [47], temperature [47, 48], glucose [47], wound pressure [16], and other parameter values during wound treatment, it can accomplish real-time tracking of wound healing. The use of conductive hydrogel in conjunction with wireless electronic technology allows for synchronous wound healing and monitoring by quickly sensing changes in wound transmission data and converting them into electrical impulses. By combining AI technology and algorithms, the intelligent conductive hydrogel dressing can also assist physicians in wound management, diagnosis, and outcome prediction. These functions have significant performance in contemporary wound treatment and can act quickly to reduce the risk of chronic wounds. Conductive hydrogel dressings that can monitor and promote healing will therefore help to advance intelligent medical care and ongoing chronic refractory wounds management.

## Materials Selection of Conductive Hydrogel Dressings

Conductive hydrogel dressings can be categorized according to the conductive components that endow them with electrical conductivity. The physical, chemical, and biological properties of various conductive materials allow them to react differently to various environmental factors and create several kinds of conductive hydrogel dressings for wound monitoring. The intrinsic material properties of constituent components exert a substantial influence on wound healing efficacy, with particular relevance to the functional optimization of conductive hydrogel systems. As a result, we may select the right materials according to the real requirements for wound healing. The benefits, drawbacks, and associated selections of various conductive materials for wound monitoring will be discussed in this section **(**Table 1**)**.

**Table 1:** Conductive materials and their advantages and disadvantages of conductive hydrogels for wound monitoring applications

Classifications	Material	Advantage	Disadvantage
Inorganic nanoparticles	CNTs Graphene and its derivative MXenes Metallic materials	Wide sensing range Excellent optical properties Excellent mechanical stability Good compatibility with wound monitoring systems Monitoring biomarkers Faster signal transmission Lower energy consumption Diversified performance Eco-friendly Broad-spectrum antimicrobial	Be prone to reunions Vulnerable oxidation High cost Poor long-term stability Vulnerable oxidation
Conductive polymers	PPy PANI PEDOT	Excellent absorbance characteristics Improve cell activation Simple to make Biomolecular monitoring Monitoring of environmental changes Multimodal monitoring Diversified performance Monitoring biomarkers	Be prone to reunions Be prone to reunions Be prone to reunions
Other materials	Ionic conductive materials	Low cost Good biocompatibility	Poor long-term stability Complex preparation

### Inorganic Nanoparticles

Conductive hydrogel dressings are mostly prepared using inorganic nanoparticles, carbon nanotubes (CNTs), graphene and its derivatives, MXenes, and metal nanoparticles are the most common types of inorganic nanomaterials.

#### CNTs

CNTs have a huge aspect ratio and conductivity comparable to metals. This unique structure significantly increases the material’s conductivity by allowing the composite conductive hydrogel to build an efficient conductive network even with a modest CNTs content [49–53]. When paired with the hydrogel matrix, CNTs’ exceptional optical and electrical qualities, exceptionally high tensile strength, and favorable compatibility with device manufacture allow them to create high-performance conductive hydrogels. CNTs are typically appropriate for wound monitoring due to their broad sensing range, high sensitivity, and good environmental tolerance [50, 54]. Nevertheless, CNTs are poorly dispersed in a variety of solvents and polymer matrices, their conductivity decreases and conductive hydrogels formed for wound monitoring that have limited sensitivity and delayed signal output. CNTs are uniformly distributed throughout the hydrogel network to guarantee their high conductivity, conductivity signal sensitivity, and strain range by adding amphiphilic sodium dodecyl sulfate, montmorillonite, and other CNTs stabilizers and dispersants [48, 49, 55].

#### Graphene and Its Derivatives

In a similar vein, graphene’s conductivity efficiency is far better than that of conventional conductive metal materials, allowing for faster signal transfer and less energy usage in conductive hydrogel dressings. Due to its high surface energy, graphene is also prone to agglomeration, which can impact its conductivity and composite effect with other materials [56]. Compared with graphene, oxidized graphene (GO) possesses additional oxygen-containing functional groups including hydroxyl, epoxy, and carboxyl groups on its surface and edges. These functional groups endow GO with excellent hydrophilicity and dispersibility, enabling it to be easily composited with other materials. Moreover, they effectively prevent agglomeration and contribute to the formation of a stable conductive network [40, 57, 58]. Their conductivity can be somewhat modified by varying the kind, amount, and distribution of these functional groups. GO’s conjugated structure is yet lost during the oxidation process, which results in a considerable conductivity drop in comparison to graphene. Many barriers to the wound monitoring signal output arise from its conductivity, which is typically only around 1/1000 to 1/100 of graphene and even shows insulation at high oxidation levels [56]. Reduced oxidized graphene (rGO) restores part of its conjugated structure, positioning its conductivity and dispersibility between those of graphene and GO. This characteristic endows rGO with excellent applicability in the wound monitoring field [16].

#### MXenes and Metal Nanoparticles

Additionally, MXenes are two-dimensional (2D) compounds made of carbonitrides and carbides of transition metals. They are particularly environmentally benign owing to their large specific surface area, metal-grade conductivity, abundance of functional groups, and low environmental effects during production and application procedures [59–61]. Its use in signal sensing for wound monitoring is limited due to its high production cost and susceptibility to oxidation [29, 62]. The exceptional electrical conductivity inherent to metal nanoparticles ensures optimal charge transfer efficiency within hydrogel-based wound monitoring systems, enabling rapid electrochemical signal transduction [52, 63, 64]. At suitable concentrations, metallic materials, such as silver nanoparticles can kill germs and promote wound healing, but their conductivity efficacy is limited by their tendency to oxidize, expensive cost of production, and uneven dispersion [28, 65].

In conclusion, although inorganic nanomaterials feature small particle sizes, complex preparation procedures, large specific surface areas, and sometimes present challenges in dispersion, researchers have proposed suitable solutions. Moreover, based on their high electrical conductivity and wide sensing range, inorganic nanomaterials are better suited for signal output in wound-monitoring sensors.

### Conductive Polymers

Conductive hydrogel dressings also contain conductive polymers, for example, polypyrrole (PPy), polyaniline (PANI), and poly (3,4-ethylenedioxythiophene) (PEDOT).

#### PPy

As a consequence of its conductive qualities, good stability, and exceptional absorbance characteristics in the near infrared (NIR) region, PPy is regarded as the most researched bioorganic conductive polymer [66–69]. Attributable to its ability to promote cell adhesion, proliferation, and differentiation and its comparatively high conductivity under physiological settings, it is very helpful when making conductive hydrogel dressings. By increasing cell activity, PPy can be added to the hydrogel to improve wound healing. It can also send bioelectrical signals to track the healing process [70].

#### PANI

PANI is frequently utilized in different electronic components production owing to its superior conductivity, easy access to raw materials, and straightforward manufacturing method [52, 71, 72]. Nevertheless, external factors such as temperature and humidity can potentially exert an influence on its electrical conductivity. The inherent gas adsorption capacity and electrochromic functionality of PANI enable its potential integration into wound status monitoring systems through electrochemical signal transduction mechanisms, thereby expanding its applicability in advanced biomedical sensing platforms [37, 73].

#### PEDOT

PEDOT is chemically sensitive, and its electrical properties (for instance, resistance, capacitance, etc.) change as it comes into contact with specific gases or liquids. It is possible to detect chemicals by recognizing these changes [74–76]. After binding with biomolecules such as enzymes and antibodies, PEDOT can be employed to detect biomarkers in organisms, including blood glucose and proteins. Designing conductive hydrogel dressings for wound monitoring is made easier by its high conductivity and strong biocompatibility [47, 56, 77].

Collectively, conductive polymers have strong environmental resilience and biocompatibility. Despite their inherent lack of mechanical strength, they are quite processable and can be mixed chemically or physically with non-conductive polymers to alter their relevant properties and increase their suitability for wound dressings. Since conductive polymers offer superior electro-optical qualities, they are more suited for various wound healing and monitoring situations.

Generally speaking, each of the conductive materials utilized to create conductive hydrogels has unique properties. For instance, metal- and carbon-based compounds cannot break down in vivo, while inorganic nanomaterials are hard to disperse. They should be thoroughly assessed before being added to wound dressings for clinical use as their size, structure, surface function, or porosity may be cytotoxic, however, their small particle size, superior conductivity, and sensitive sensing capabilities make them ideal for wound monitoring applications. Although conductive polymers exhibit high biocompatibility, their ability to facilitate wound healing and maintain the continuity of wound-monitoring procedures can be impeded. As their slow degradation and consumption under physiological conditions, along with a possible decline in conductivity over time. Currently, the majority of research uses conductive hydrogel dressings composed of a single conducting substance. Conductive hydrogel dressings may not be enough to completely monitor the entire healing process as they are typically only effective in one stage of wound healing. Therefore, future research should focus more on the cooperative application of conductive materials, the combined use of conductive materials can compensate for the absence of material characteristics to support wound-healing cell activity and track the healing process. Different materials are appropriate for various wound monitoring signal output scenarios due to their distinct features. We will then concentrate on the specific use of various wound types, as well as the monitoring method and signal output classification of conductive hydrogel utilized for wound monitoring.

## Wounds Monitoring Mechanisms

**Fig. 3:**
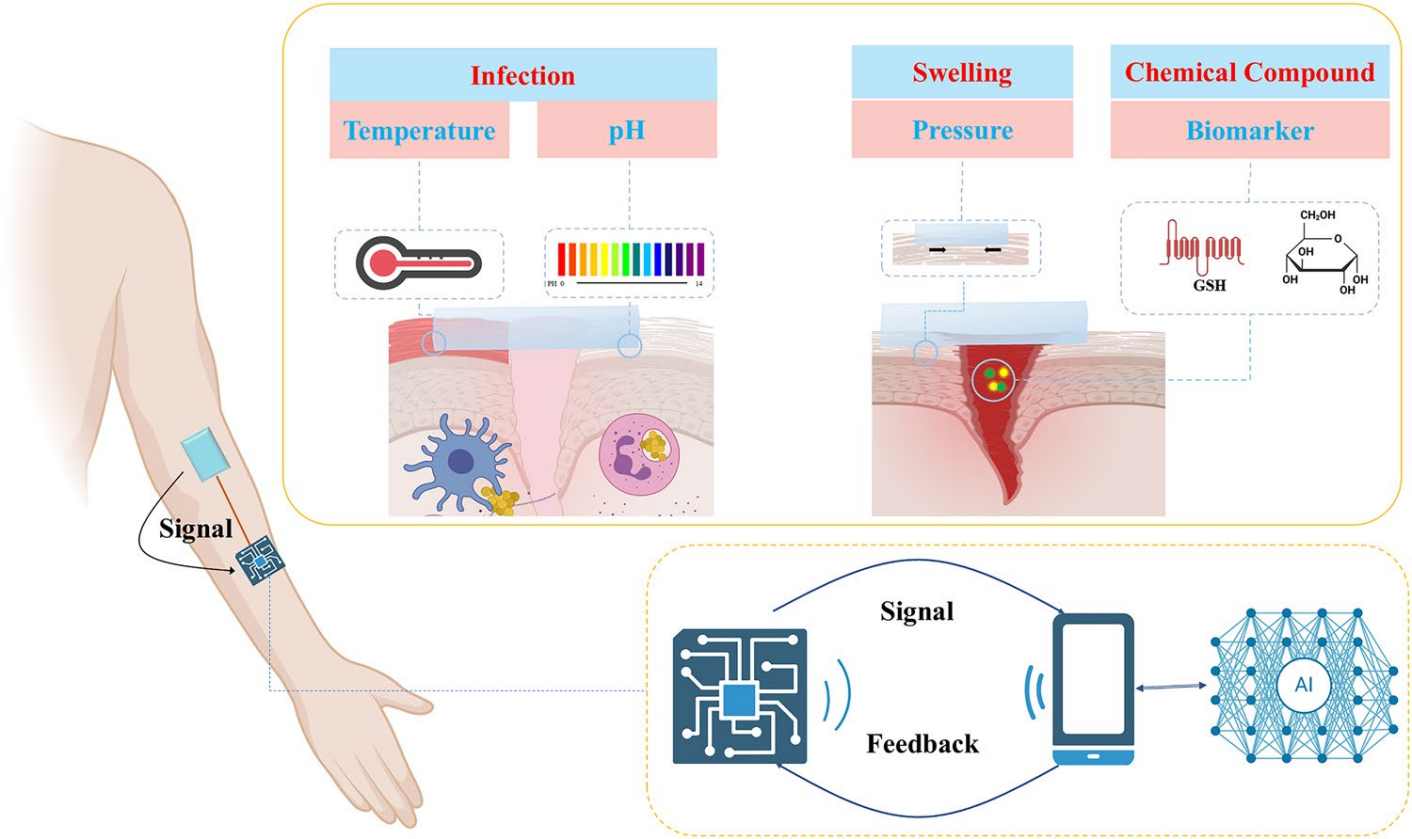
Schematic illustration of conductive hydrogel dressings in different wounds monitoring and signal transmission

In recent years, there has been a substantial surge in research dedicated to the development of intelligent dressings, driven by the escalating demand for integrated sensors for wound monitoring and treatment. For integrated wound monitoring and treatment, the signal output kinds of conductive hydrogel dressings can be broadly categorized into thermal physiological signal-temperature, chemical physiological signal-pH and glucose, mechanical physiological signal-pressure and other physiological signals (Fig. 3). At present, the sensing types of conductive hydrogels used to monitor wound-related physiological signals mainly include infrared imaging, colorimetry and resistance sensors. Infrared imaging is based on the radiation of infrared rays by objects, which are converted into electrical signals by infrared detectors to generate images reflecting temperature distribution. The colorimetric method determines the concentration of the substance by measuring its absorbance using colorimetric reactions and a spectrophotometer based on its absorption characteristics of light. Resistance sensors are based on the principle that material resistance changes with physical quantities (pressure, temperature, etc.). By measuring the circuit, the resistance changes are converted into electrical signals and processed to obtain relevant physical quantity information (Table 2). This section systematically delineates the clinical implementation paradigms and quantitative characterization methodologies for multimodal output signals generated by conductive hydrogel-based dressings during dynamic wound microenvironment monitoring.

**Table 2:** Conductive hydrogel for monitoring wound healing and its characteristics

Physiological signal	Monitoring signal	Material	Monitoring method	Characteristic	Wound type						
Thermal physiological signal	Temperature	CNTs MXenes	Infrared illumination [48] Colorimetry [78] Resistance variation [29]	Real-time monitoring High sensitivity Non-invasive Limited scope of application Short-term usage	Diabetic ulcers Pressure ulcers						
Chemical physiological signal	pH Blood glucose	Ion conductive materials PANI PPy	Colorimetry [79] Resistance variation [23, 70]	Real-time monitoring High sensitivity Instability Costly	Diabetic ulcers Pressure ulcers						
Mechanical physiological signal	Pressure	Ionic conductive materials	Resistance variation [80] Colorimetry and resistance variation [81]	Real-time monitoring Quantitative monitoring Multiparameter monitoring Costly	Diabetic ulcers Pressure ulcers Articular wounds						
Other physiological signals	Pain signal Skin integrity	Metal nanoparticles	Colorimetry [82] Resistance variation [77]	Real-time monitoring Quantitative monitoring Unstable signals Costly	Diabetic ulcers Pressure ulcers						

### Thermal Physiological Signal—Temperature

**Fig. 4:**
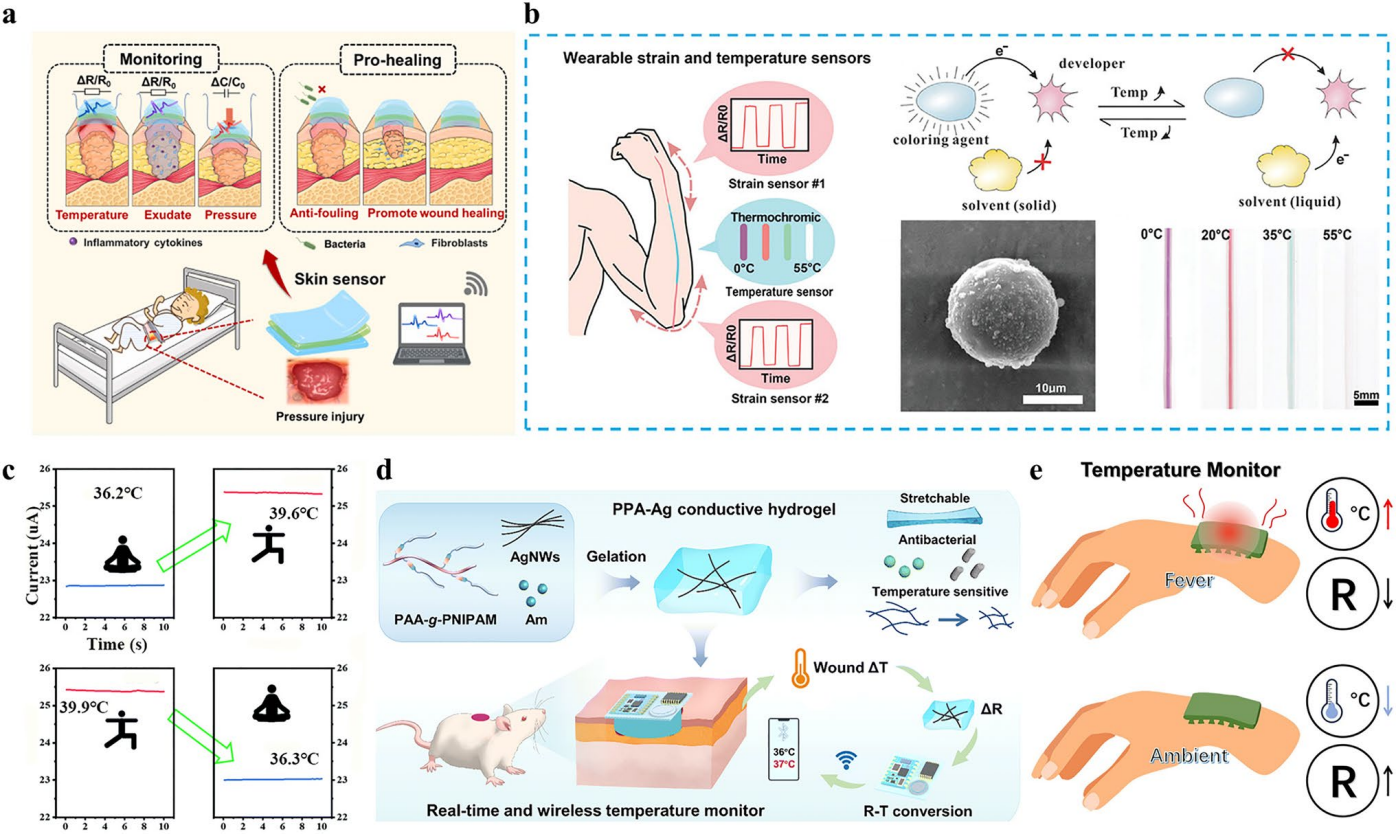
Mechanism of temperature monitoring as a wound thermal physiological signal for conductive hydrogel dressings. **a** Wound temperature changes affect hydrogel temperature changes, allowing for remote monitoring of the wound. Reproduced with permission from Ref. [18]. Copyright 2022, Elsevier. **b** Schematic diagram of a hybrid fiber for dual strain and temperature sensors. Reproduced with permission from Ref. [90]. Copyright 2024, Wiley–VCH. **c** Design of conductive and temperature-sensitive hydrogel dressing strategies integrated with a wireless Bluetooth module for real-time wound temperature monitoring. Reproduced with permission from Ref. [29]. Copyright 2024, Elsevier. **d** Continuous temperature monitoring is realized by resistance change at different temperatures. Reproduced with permission from Ref. [28]. Copyright 2023, Royal Society of Chemistry. **e** Current changes before and after “artificial fever”. Reproduced with permission from Ref. [91]. Copyright 2021, Royal Society of Chemistry

Inflammation, vasodilation caused by inflammatory chemicals, and immunological reactions can all cause the skin wound tissue temperature to increase throughout the healing process. This temperature increase is roughly 1.5 °C greater than that of normal skin (Fig. 4a) [18, 83]. Furthermore, an infection may cause the wound temperature to rise even more [84]. Consequently, closely monitoring the temperature during the wound healing process proves to be of great significance and advantage. Through various signal outputs, it can not only closely comprehend the wound healing status but also promptly address unfavorable situations containing wound infection [85]. Numerous techniques, including colorimetric sensors, infrared thermometers, and electronic temperature-responsive dressings, have been developed for temperature monitoring [23, 86, 87].

A photosensitive imaging device quickly records the radiant energy and temperature distribution released from the human body by converting the infrared band created by an object’s thermal radiation into a recognizable temperature pattern. Infrared thermography is a quick, non-contact, and non-invasive technology. The measurement of test results is applicable in clinical practice for detecting abnormal blood flow or inflammation in subcutaneous tissue [88]. Using CNTs as a conductive substance, Shen et al. [48] created a detecting wound dressing that was applied to the mice’s wound site. Infrared thermography was used to measure the wound site temperature in real time. Even if there was only a 2–3 °C temperature difference, the wound status could be accurately ascertained by comparing it to the temperature of the uninjured area in the control group. The thermometric precision at the wound interface is validated through correlated electrochemical impedance reduction and upregulated pro-inflammatory cytokine expression profiles demonstrated via immunohistochemical quantification. This sensor’s high sensitivity and broad sensing range allow it to detect even minute temperature changes.

The primary method for creating colorimetric temperature sensors involves mixing temperature-responsive materials with dyes or noble metal nanoparticles (NMNPs). Based on localized surface plasmon resonance (LSPR), these materials exhibit visible color via significant absorption in the visible light spectrum. The theory relies on the shift in the absorption spectrum, which can be attributed to either a significant change in the absorption coefficient or a change in the energy level difference of the system following identification (Fig. 4b) [89, 90]. Using pre-gel for sequence replication and populating colloidal crystal templates, Wang et al. [78] developed a structural color Li^+^-ionic hydrogel as an intelligent wound-healing patch. In addition to promoting tissue healing and cell proliferation in wounds, the VEGF-coated hydrogel patch enhanced detection accuracy and dependability by integrating structural color sensing with electrical signal monitoring. The hydrogel patch exhibited clear color changes from red to green when the wound’s temperature rose, put another way, the alterations in the structure and the hydrogel patch color enabled the intuitive assessment of the wound’s temperature.

Apart from colorimetric sensors and infrared thermometers, resistance type temperature sensors can also be used to track temperature variations at the wound site, are usually composed of a stretchable polymer hydrogel integrated with conductive materials. The fundamental building block of the sensing mechanism is thermosensitive materials. As the temperature increases, the resistance of thermosensitive materials will change with alterations in their geometric shape or transmission mechanism. Different resistance change signals are output to track the temperature change at the wound (Fig. 4c) [91]. Ma et al. [29] added MXenes to the hydrogel system, which greatly increased the hydrogel’s heat conductivity. Rapid temperature response is ensured by the MXenes network’s high thermal conductivity, where the thermal disturbance induced by temperature rise supplies energy for more electronic transitions. The conductivity of MXenes increases with temperature under thermal excitation since the rise in carrier concentration. The resistance of the conductive hydrogel sensor that measures the wound temperature is inversely proportional to the temperature and conductivity and resistance are inversely related. The resistance signal drops in response to infection or other conditions at the wound site, and it is possible to monitor the wound temperature in real time by adjusting the output resistance. The goal of integrating therapy and monitoring was fully achieved by the conductive hydrogel created in this work, which monitored temperature and performed well in photothermal treatment (Fig. 4d). Wang et al. [62] also used resistance signal output to react to changes in the local temperature of frostbite wounds. This MXene-based conductive composite hydrogel sensor is not merely applicable as a dressing to accelerate the healing of frostbite wounds. It can also be employed to monitor, collect, and transmit electrical signals in real-time. Furthermore, it can transmit human electrophysiological signals to mobile devices through Bluetooth wireless technology. This enabled medical professionals to precisely evaluate and remotely administer treatment for patients’ conditions. Silver nanowires (AgNWs) with antibacterial and sensing capabilities were added to the three-dimensional (3D) conductive hydrogel network by Jiang et al. [28], who also linked the conductive hydrogel matrix to the wireless transmission module. The hydrogel resistance changed in response to the change in wound temperature, and the intelligent device wirelessly received the temperature change. To achieve real-time wireless monitoring of wound temperature and aid in early infection diagnosis, they measured the hydrogel’s resistance during a temperature cycle between 25 and 42 °C. The results indicated that the resistance had minimal fluctuations and good stability over 14 days (Fig. 4e). Dang et al. [92] conducted another research to investigate the resistance change of hydrogels under more subtle temperature variations, taking into account the narrow variation range of human body temperature. It is possible to utilize the Fe^3+^ and carboxymethyl cellulose conductive hydrogel sensor as a “fever indicator” to anticipate wound infection and gauge the severity of infection since it can detect temperature changes at the wound with more accuracy.

Infrared thermographic analysis serves as a critical validation tool for thermoresponsive conductive hydrogel systems, providing real-time visualization of localized thermal fluctuations in wound beds and their concomitant electrochemical signal modulations, thereby establishing multimodal sensing platforms through temperature-dependent chromatic transitions in integrated colorimetric sensors. Using intuitive observation, these two approaches are quick and easy, but they have little reference value and are quite subjective. The subject of wound temperature monitoring makes extensive use of resistance temperature sensors. Temperature changes can be measured as resistance changes utilizing the idea that conductivity changes with temperature and produces various resistance changes. These resistance changes can then be sent to mobile devices via wireless Bluetooth. This remote monitoring system improves clinical efficiency by enabling real-time tracking of wound healing progress, thus reducing unnecessary follow-up visits and optimizing time management in patient care. However, as the wound microenvironment is a dynamic system including several variables like pressure, temperature, inflammatory factors, pH, and blood oxygen, current research that depends only on temperature as a single metric to track wound health has serious limits. The pathophysiological process cannot be well reflected by a single indicator, which might result in inaccurate assessments of the genesis and condition of wounds. To accomplish multidimensional and multi-parameter monitoring, more investigation is required.

### Chemical Physiological Signal

#### pH

The pH of healthy skin ranges from 4.8 to 5.7, which is somewhat acidic [93]. Exudate will form at the site of the wound when the skin is damaged, and as a result of many pathological events, including inflammation, collagen creation, and angiogenesis, the pH value of the exudate will vary. Blood vessel constriction in the wound’s local tissue during the early inflammatory stage may result in an inadequate blood supply, which in turn may lead to inadequate oxygen and nutrition. Conversely, lactate and CO_2_ rise due to glycolysis, which eventually lowers pH. An acidic environment can enhance wound healing, stimulate fibroblast proliferation, and boost the supply of oxygen. During the proliferative phase of wound healing, pathogenic infection triggers ECM degradation and ammonia release, elevating local pH to 7.15–8.90. This alkaline microenvironment promotes bacterial colonization through pH-sensitive biofilm formation pathways [46, 94]. Monitoring pH variations throughout the wound healing process is therefore extremely important from a therapeutic standpoint. Based on the present trend of pH changes, in addition to quickly evaluating the wound’s health, it may also provide clinical physicians with an early warning for the best course of treatment.

**Fig. 5:**
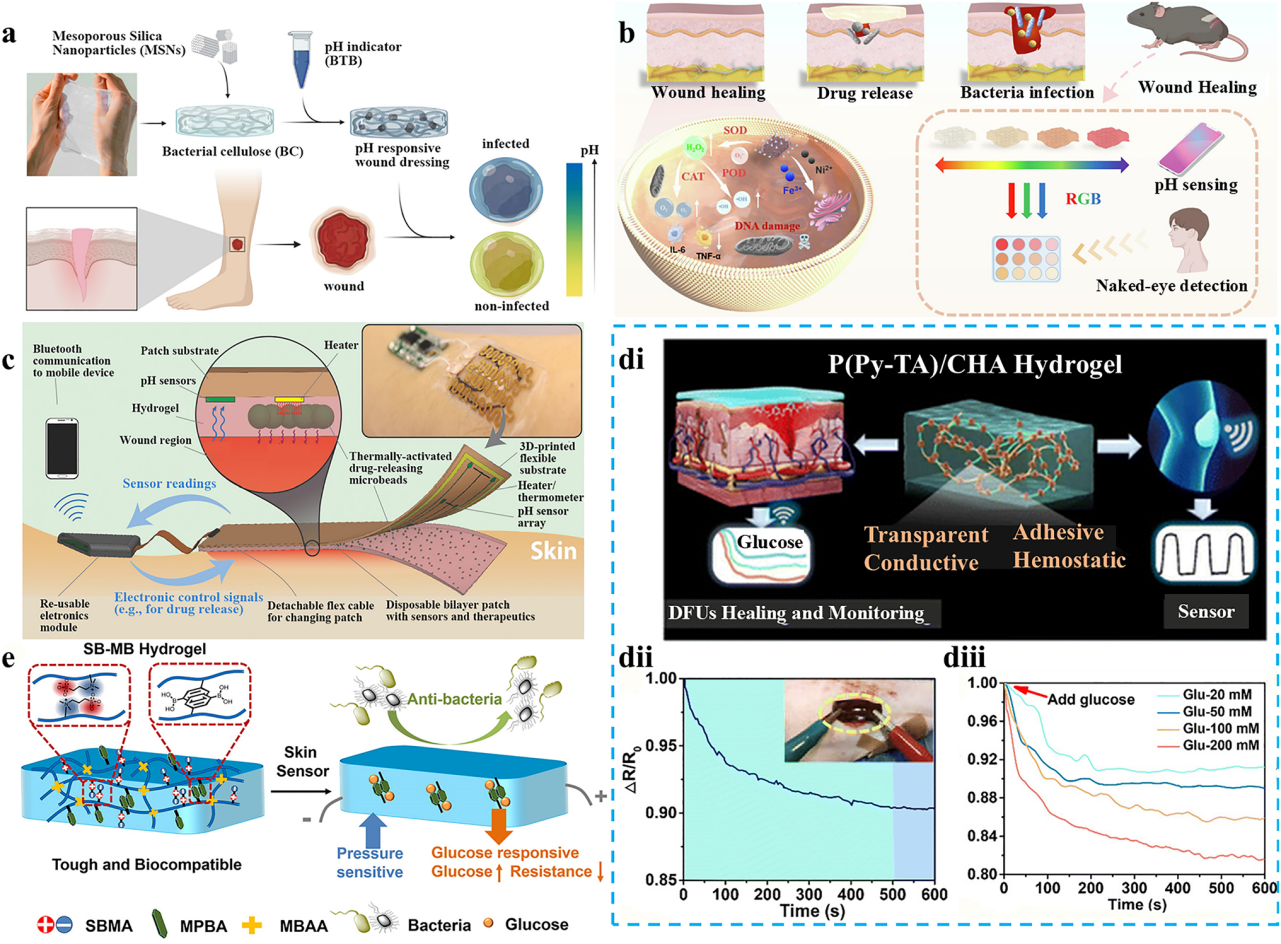
Mechanism of wound chemical physiological signal monitoring for conductive hydrogel dressings. **a** A pH-responsive nanocomposite wound dressing was obtained by impregnating bacterial nanocellulose with mesoporous silica nanoparticles loaded with pH-responsive dye. Reproduced with permission from Ref. [21]. Copyright 2024, Elsevier. **b** Diagram of the use of hydrogels for pH monitoring and treating bacterium-infected wounds. Reproduced with permission from Ref. [79]. Copyright 2024, Elsevier. **c** Schematic and conceptual diagram of an automated smart bandage. The bandage consists of a series of flexible pH sensors and a flexible heater for triggering a heat-responsive drug carrier containing antibiotics. The electronic module also allows for wireless communication with computers and smartphones. Reproduced with permission from Ref. [23]. Copyright 2018, Wiley–VCH. **di** The applications of P(Py-TA)/CHA hydrogel patch in DFUs healing and glucose monitoring. **dii** The resistance response curve to glucose at the wound in real-time. **diii** Relative resistance change curve of P(Py-TA)/CHA hydrogels with glucose concentration change from 20 to 200 mM. Reproduced with permission from Ref. [70]. Copyright 2012, Royal Society of Chemistry. **e** Molecular design and schematic of the SB-MB skin sensor, which enables continuous monitoring of blood glucose at the wound site. Reproduced with permission from Ref. [96]. Copyright 2021, Elsevier

Currently, for the monitoring of wound pH values, the majority of methods rely on visual inspection through colorimetric control. This involves adding pH-responsive substances to wound dressings. These substances trigger color changes, allowing for the detection and perception of the acid–base conditions within the wound’s microenvironment (Fig. 5a) [21]. Zou et al. [79] built an intelligent diagnosis and treatment hydrogel system by mixing FeNi metal organic framework (MOF) and pH indicator (phenol red) into the hydrogel. Through color change, the hydrogel dressing gave real-time wound data, including the degree of bacterial infection and the situation of wound healing. The dressing may do digital remote diagnosis and therapy when it was connected to the image acquisition device by monitoring the pH value of the wound site in real time and drawing a standard curve between RGB values and pH. This helped with precise intervention to achieve optimal wound healing. By offering thorough monitoring and action based on precise and timely wound information, this technology improved wound care (Fig. 5b). Voltage type pH sensors can also monitor changes in pH at the wound site. To monitor the pH value, the conductive hydrogel’s working principle involved protonating and deprotonating the working electrode in an acidic and alkaline environment. This charge accumulation resulted in a voltage output. By regularly checking the wound’s pH, Mostafalu et al. [23] created a conductive hydrogel based on alginate/PANI that can identify bacterial infections in situ and administer antibiotics locally when necessary. According to the feedback data from the pH sensor, when the bacteria entered the lag phase, the pH value dropped to 6.5. Cefazolin was automatically released into the wound area by the conductive hydrogel bandage when the pH reached this level. The electronic patch was equipped with a wireless Bluetooth Low Energy (BLE) module, which enabled remote drug-stimulated release and the reading of data from the pH sensor. Additionally, the drug release strategy can be customized for individualized treatment. The subjective element of colorimetric comparison was avoided by using this conductive hydrogel dressing to measure the wound pH, and the quantitative monitoring data improved the accuracy and dependability of the results (Fig. 5c). At present, the conductive hydrogel dressings enable dual-mode wound pH monitoring through colorimetric visualization and voltage-based quantification. The relevant medication is then configured for release after the sensor data is sent and received via mobile electronic devices. In addition to facilitating wound healing, this wireless communication feature keeps patients, caregivers, and physicians in remote contact during the whole wound care procedure. Nevertheless, there are drawbacks such as low drug release precision, inadequate multimodal fusion in data algorithms, and significant subjectivity in color comparison. Further research is needed to optimize materials, data fusion, algorithms, and other aspects.

#### Blood Glucose

Beyond pH dynamics monitoring, glycemic variability during tissue repair emerges as a critical biomarker in diabetic wound management, with sustained metabolic fluctuations and high blood glucose levels directly influencing angiogenesis efficacy and ECM remodeling processes. When the conductive hydrogel contacts glucose of different concentrations, the hydroxyl group selectively reacts with glucose, generating a cyclic borate ester anion, which leads to free water extrusion in the hydrogel and the ion concentration increases, and ΔR/R_0_ presents a regular trend of change. Thus, conductive hydrogel glucose sensors can be fabricated using aminophenylboronic acid-modified nanocomposites. The wide detection range, high response stability, and outstanding glucose-response sensitivity of the sensor are all attributed to the selectivity of aminophenylboronic acid toward glucose [95]. Through the promotion of intercellular signal transmission, angiogenesis, collagen deposition, and wound infection control, Liu et al. [70] created a conductive hydrogel patch doped with PPy nanofilaments that had adjustable transparency and could effectively promote diabetes wound healing. This hydrogel patch allowed for real-time monitoring of the glucose level at the wound, which offered a trustworthy reference for diabetic wounds management in clinical applications. When it came into contact with gradually increasing glucose concentration, the resistivity exhibited a declining trend with regular changes and was consistent with the results detected by commercial glucose meters (Fig. 5di-diii). In a similar manner, Guo et al. [96] created a zwitterionic conductive hydrogel dressing by decreasing resistance when the blood glucose level at the lesion rose. This integrated system enabled simultaneous real-time glycemic tracking at wound sites and telemedicine-enabled remote surveillance through bidirectional human–machine interfaces, establishing a closed-loop monitoring framework for diabetic wound management (Fig. 5e). Presently, the conductive hydrogel dressing that is intended to track blood glucose levels may efficiently track wound glucose levels in real time by detecting changes in electrical resistance, and it can also aid in the healing of chronic wounds. The blood glucose monitoring of diabetic wounds does not have visible colorimetric analysis, despite the creation of the high transparency conductive hydrogel dressing. Additional research can improve the linked sectors.

### Mechanical Physiological Signal—Pressure

The development of hematoma and edema during the inflammatory phase of wound healing causes the pressure to rise quickly and reach its peak, the pressure then gradually falls during the proliferative phase as the hematoma is absorbed, granulation tissue is filled, and epithelial cells migrate, the pressure is stabilized during the remodeling phase by collagen remodeling and epidermal maturation, and only the scar hypertrophy site may have high local tension. Pathological factors involving infection, uncontrolled edema, and hematoma can cause abnormally high pressure, while external interventions such as pressure bandaging and unplanned pressures with positional compression and tight external fixation can result in less blood and nutrients reaching damaged tissues [97]. Furthermore, the cytokine imbalance in the wound exudate, characterized by an increase in pro-inflammatory mediators and a decrease in tissue-repair factors, impairs the immunomodulatory capacity of the wound. This impairment, in turn, makes the wound more susceptible to pathogenic colonization. In conclusion, there is a vicious cycle of infection and increased exudate that results in tissue damage from prolonged stress [98, 99]. It is crucial to focus on the pressure on the wound site to target and promptly treat pressure wounds, which are more common in patients with diabetes and those who have been bedridden for an extended period. Continuous interface pressure monitoring at wound sites is therefore essential, as wounds can be treated specifically and promptly. Patients with pressure ulcers and articular wounds are more susceptible to secondary injuries caused by external forces (such as stretching or compression of the wound) or exercise during the wound healing process, which may seriously hinder wound healing [100]. Wound healing will be significantly improved by early identification and prompt treatment of aberrant wound healing [80].

**Fig. 6:**
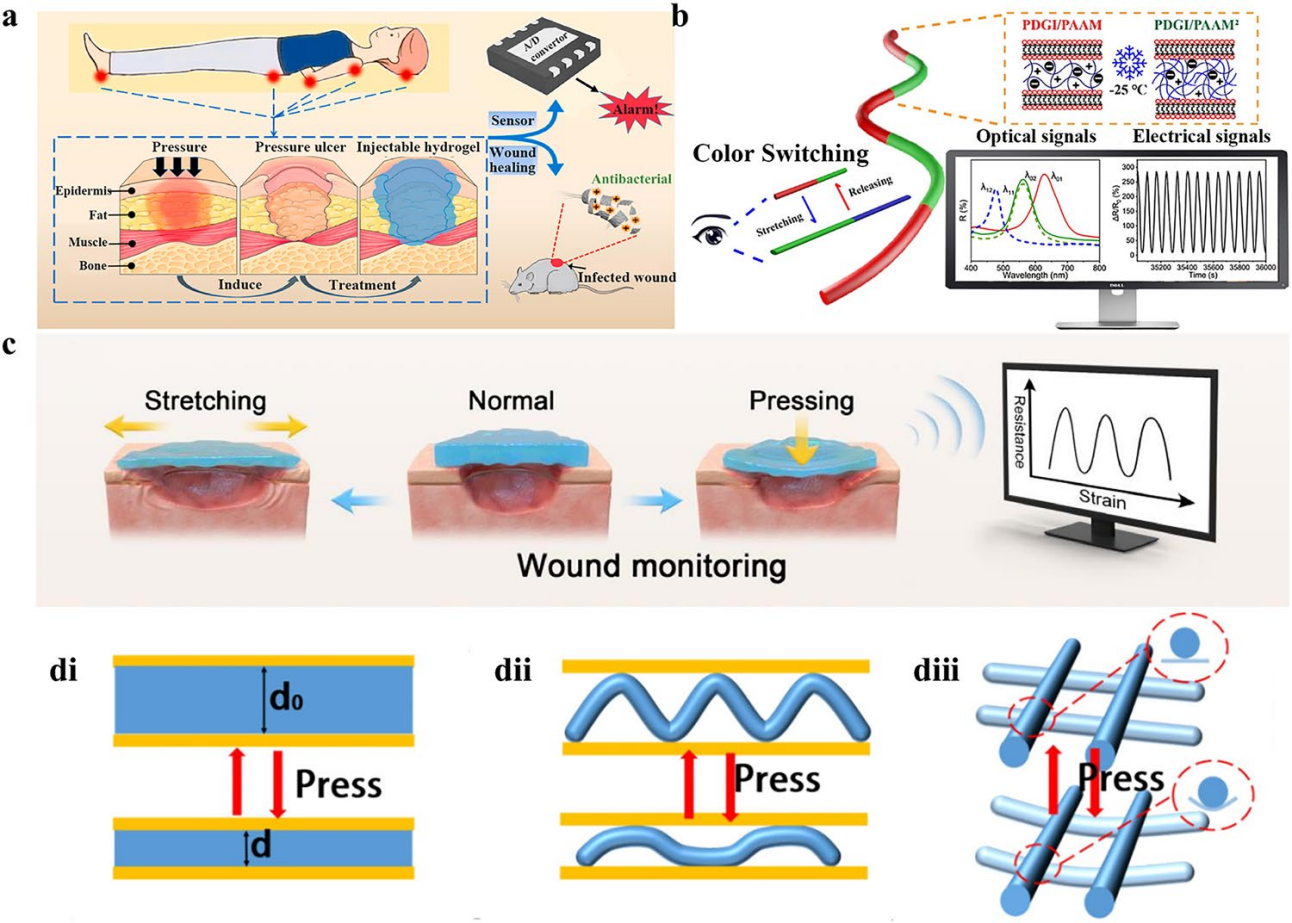
Mechanism of pressure monitoring as a wound mechanical physiological signal for conductive hydrogel dressings. **a** 3D hybrid stent monitors the pressure exerted on the wound in real time to prevent the formation of pressure sores and has antimicrobial moieties to promote the healing of infected wounds. Reproduced with permission from Ref. [16]. Copyright 2022, Elsevier. **b** Schematic illustration depicting the structure and application of the HSCEF for the interactive wearable sensor. Reproduced with permission from Ref. [81]. Copyright 2022, American Chemical Society. **c** The detection of pressure ulcers is achieved by utilizing the stress changes caused by stretching and pressing of the wound. Reproduced with permission from Ref. [80]. Copyright 2023, Elsevier. **di**–**diii** Schematic representation of the pressure sensing mechanism of conductive hydrogels with block structure, aligned fiber structure and crossed fiber structure. Reproduced with permission from Ref. [17]. Copyright 2025, Elsevier

By transforming the mechanical signal (pressure) during the wound healing stage into a readable electrical signal (mostly resistance), the conductive hydrogel-based sensor can monitor human health (Fig. 6a) [16]. A conductive hydrogel sensor array based on ionic liquid was created by Wang et al. [101] that can detect strain or force in 3D and reflect the force on the wound surface by altering the output resistance. As a result, 3D sensor arrays can be affixed to the skin’s surface to track wound healing in real time. Moreover, 3D arrays of various sizes and locations can be created according to the location and size of the wound, allowing for more individualized treatment. Ge et al. [102] designed a conductive hydrogel with a PNAGA/AgNW pressure sensing layer. To assess the mechanical strain on the wound, direct the patients’ health exercises, prevent secondary fractures of the wound, and lessen the formation of scars, the conductive hydrogel PNAGA/AgNW layer’s resistance is highly sensitive to the applied strain. This resistance change allowed for the identification of different strains (tension or compression). Additionally, Zhang et al. [80] created a conductive hydrogel with ionic liquid as a pressure sensor to track pressure-induced tissue damage in diabetic wounds. The sensor’s resistance dropped when the hydrogel was pressed firmly. According to the output resistance signal, it became feasible to evaluate the pressure exerted on the wound and the status of wound healing (Fig. 6c). By adding ionic liquid, Zhang et al. [81] created an interactive electronic optical fiber gel sensor with isomeric structural color that can quickly and synchronously produce photoelectric dual signals under stress. The diverse color gradually turns blue as the applied tension rises. This conductive gel’s color may transition between orange and cyan, green and blue, and cyan and blue dynamically. The final color turns blue-purple as the strain increases further. Stable and sensitive electrical impulses are output synchronously. According to the results, this variable color can provide both qualitative and quantitative stress and strain results by switching feedback information and relative resistance changes across the visible band (675–450 nm). This offers twofold protection and is essential for tracking external damage during the wound healing process (Fig. 6b).

Conductive hydrogel dressings can achieve remote sensing and analysis of pressure changes based on the pressure measurement at the wound by reading the transducer signals (Fig. 6di-diii). By detecting signs including wound temperature, pressure, and exudate, Liu et al. [18] created a skin sensor that allowed for continuous real-time monitoring of inflammation, swelling, and suppuration as well as aiding in the healing of pressure ulcers. It can stop irreversible stage (III or IV) damage from getting worse and show the reversible stage (I or II) of wound healing. Three factors (E: exudate, P: pressure, and T: temperature) were simultaneously monitored by the system, which was made up of two ion resistance sensors (upper hydrogel and lower hydrogel) and a parallel plate capacitive sensor (double hydrogel). The 3D color map of the bottom sensor that was in direct touch with the pressure ulcer displayed responses to three different forms of stimulus information, however, the upper sensor can only monitor temperature and pressure owing to the barrier effect of the intermediate isolation layer. The double hydrogel was a parallel plate capacitive sensor that was not affected by exudate or temperature. This zwitterionic conductive hydrogel dressing can simultaneously monitor and distinguish multiple parameters, which is conducive to home care and rehabilitation of pressure sore patients, especially for patients with sensory loss or dementia.

Monitoring pressure changes at the wound site is of great significance for patients with pressure ulcers and articular wounds, devoted care consumes resources and energy. Local pressure signals from wounds are currently turned into resistance signals by resistance sensors, which are further transformed into digital signals by converters. Medical personnel can analyze and judge pressure changes at the wound site quantitatively, even from a distance. Apart from the basic electrical signal sensor, a hydrogel sensor capable of synchronously producing photoelectric signals has also been created. Home nursing personnel and other non-professionals can benefit greatly from its ability to make direct visual assessments besides precisely detecting the pressure at the wound. It will be more useful in clinical practice to have a sensor that can monitor numerous signals simultaneously, as opposed to a conductive hydrogel sensor that simply measures the pressure at the wound. This sort of conductive hydrogel dressings also needs further research in the future.

### Other Physiological Signals

During the process of applying conductive hydrogel dressing on the injured part of the patient to promote wound healing, the doctors do not know much about the internal wound condition of the dressing, and only treat the patients according to the overall condition. Visual inspection, photography, and measurements of the wounds’ size and depth are typically used to assess and monitor refractory wounds. Frequent dressing changes are particularly bothersome if it is necessary to assess the wounds’ health. This can easily rip the unhealed wound and potentially result in secondary harm. Medical personnel can conduct reasonably accurate assessments of the wounds’ healing condition by tracking the physiological signals coming from the wound and translating them into measurable signal outputs.

#### Neural Physiological Signal—Pain Signal

**Fig. 7:**
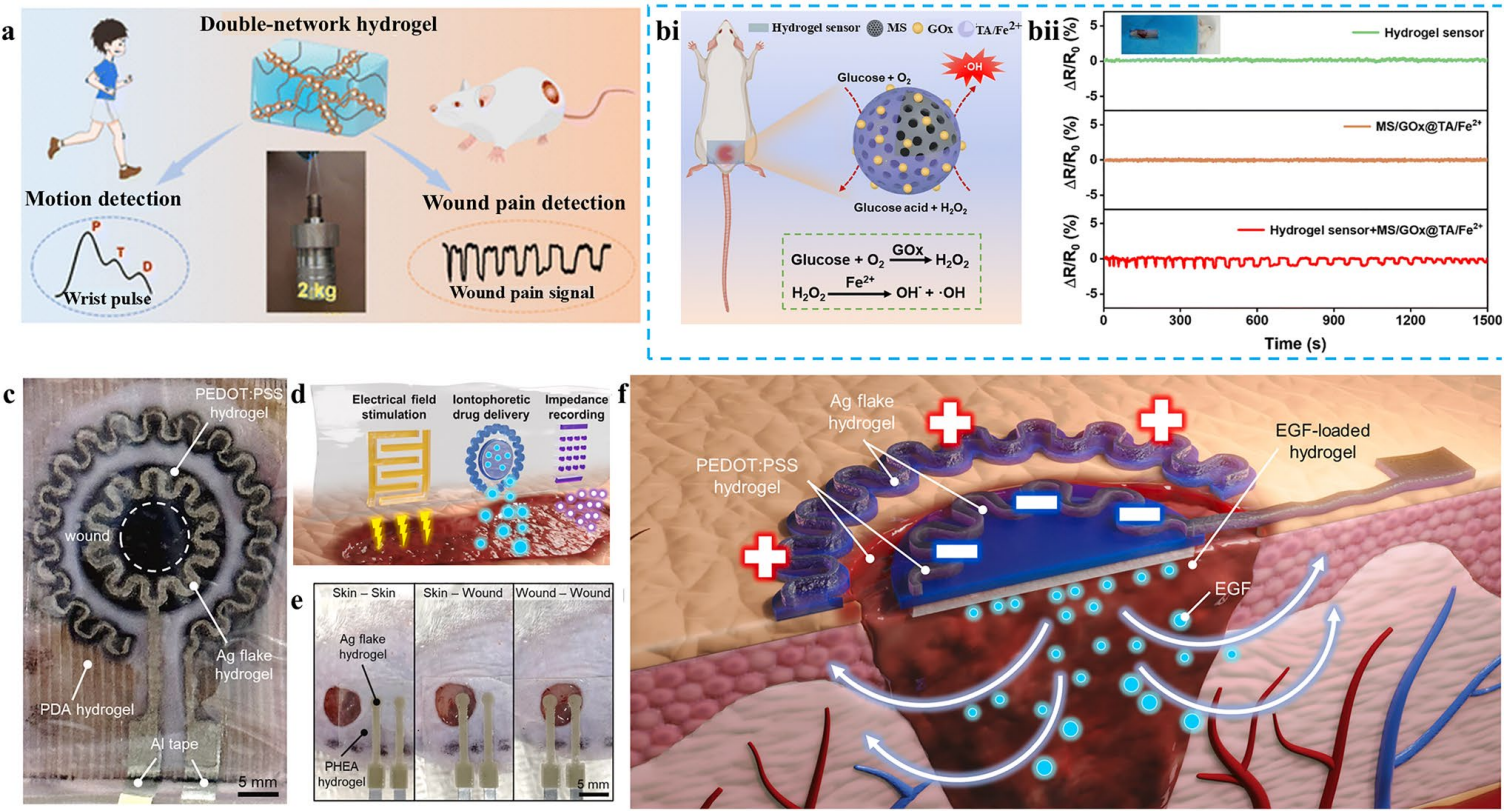
Mechanism of other wound physiological signals monitoring for conductive hydrogel dressings. **a** Schematic illustration depicting the application of the DN hydrogel for the wound pain signal detection. **bi** Schematic demonstration of in situ monitoring of pains caused by cascade reactions using a DN hydrogel sensor. **bii** Real-time resistance signals from the DN hydrogel sensor. Reproduced with permission from Ref. [82]. Copyright 2023, Royal Society of Chemistry. **c** Images of e-skin patches for iontophoretic therapy applied to mouse skin wounds. **d** Representative hydrogel patterns of e-skin patches showing each application of electric field stimulation, iontophoretic drug delivery, and impedance recording. **e** Images of impedance measurements using e-skin patches at various sites. **f** Schematic diagrams of e-skin patches for iontophoretic drug delivery. Reproduced with permission from Ref. [77]. Copyright 2025, Elsevier

Diabetic wounds are being monitored for pain perception apart from blood glucose levels. The feeling of pain gradually goes away as the wound heals. By adding metallic nanoparticles to the hydrogel system to track the minor pain caused by the diabetic wound cascade reaction, Zhang et al. [82] assessed the condition of the wound (Fig. 7a). Changes in the electrical signal at the wound site could be seen, and the ROS reaction-generated ·OH staining showed the level of discomfort there (Fig. 7bi). Initially, the resistance waveform was distinct and well-defined. As the monitoring duration is prolonged, it is broadened. This phenomenon may be attributed to the increased presence of ·OH radicals, which was indicative of heightened pain at the wound site. The waveform gradually reverted to its original sharpness. This could be ascribed to the fact that the consumption of ·OH radicals alleviates pain. The sensitivity of the conductive hydrogel sensor in tracking the pain signal at the wound was demonstrated by the resistance change during the test, which was consistent with the change in ·OH content. Changes in the wound site can be handled quickly and precisely by measuring the pain signals there and instantly notifying medical personnel (Fig. 7bii). Improving the comfort level of patients who have trouble expressing themselves verbally can help develop individualized pain relief plans, improve treatment safety and effectiveness, and increase patient satisfaction. Monitoring pain signals at the wound site is important as it can quantify the quality of healing and provide early warning of infection or complications. The majority of pain signal monitoring, however, now relies on electrical signals via resistance variations. To accomplish multidimensional monitoring, other assessment techniques can be researched in the future.

#### Structural Physiological Signal—Skin Integrity

Visual methods are frequently employed for evaluating the wound-healing process. While these techniques are undoubtedly beneficial, they can be subjective as they predominantly rely on the observer’s judgment. The resistance of biological tissues to alternating current is known as bioimpedance. The bioimpedance measurement system offers a quantitative, objective, and interference-free way to track the wound healing process to get beyond these restrictions. When cells are damaged in the wound area, ions and currents can readily flow through the cell membrane, increasing conductivity and decreasing impedance [103, 104]. By altering the impedance, one can ascertain the integrity of the skin at the wound site using this approach. Impedance was measured in three different places by Shin et al. [77] with PEDOT: PSS and silver nanosheet hydrogel: between normal skin, between skin and wound, and between wounds. Impedance readings were sorted by skin, skin wound, and wound-wound site in descending order across all frequency ranges. A 4 × 4 working electrode array and two counter electrodes were used to evaluate the impedance of the wound and the surrounding undamaged skin in a mouse wound model. The wound state characteristics of each unit in the 4 × 4 array were then determined by comparing the measured wound impedance with the reference impedance value (Fig. 7c-e). After that, a 7 × 7 grid was created from the skin area at the wound site, and linear interpolation was used to calculate the values of each cell. 2D colorimetric impedance maps were used to show these values. The wound indicated in red on the impedance diagram steadily diminished as the wound healing process advanced, suggesting that the wound was healing gradually. A novel approach to real-time monitoring and diagnosis was offered by this semi-quantitative intelligent wound monitoring procedure that used multifunctional conductive hydrogel as a wound dressing. It also offered promising potential for speeding up wound healing and enhancing patient quality of life (Fig. 7f). Research that integrates pertinent algorithm analysis with real-time monitoring of the healing process by bioimpedance mapping to speed wound healing is currently absent. To improve the intelligence and accuracy of monitoring, future research will focus on preprocessing, feature extraction, and intelligent modeling of multimodal signals involving temperature and pressure to recognize wound states, predict healing, and support clinical decisions.

Medical personnel can conduct precise assessments of wound healing by tracking physiological signals from wounds in real time, such as pain signals and skin integrity, and translating these into qualitative or quantitative single or dual signal outputs. Nevertheless, little study has been done on additional output signal processing as of yet. To enable real-time remote monitoring and treatment of the patient’s wound status, researchers should additionally connect the output signal to backend equipment for processing.

## AI-Assisted Wounds Monitoring

A new generation of intelligent wound dressings that integrate several flexible conductive sensors has evolved with the quick development of emerging flexible electronic technologies. This allows for real-time wound condition monitoring via a variety of physiological signals, comprising temperature, pH, blood glucose, pressure, etc. [9, 23, 24, 105, 106]. In addition to in situ wound sensing and on-demand treatment [107–113], in the upcoming era of precision and personalized medicine, technologies such as AI, big data, and image processing present feasible and promising approaches for developing more reliable and user-friendly intelligent wound treatment solutions [114–117]. There are many benefits to using machine learning for wound monitoring owing to its ability to handle data effectively. The effectiveness of wound monitoring is significantly increased by rapidly processing these massive amounts of data and obtaining useful information from them. Real-time and personalized wound monitoring is made possible by deep learning models’ ability to autonomously extract intricate characteristics and patterns from vast amounts of biological monitoring data, recognize various wound states with accuracy, and process and evaluate fresh incoming data in real time.

**Fig. 8:**
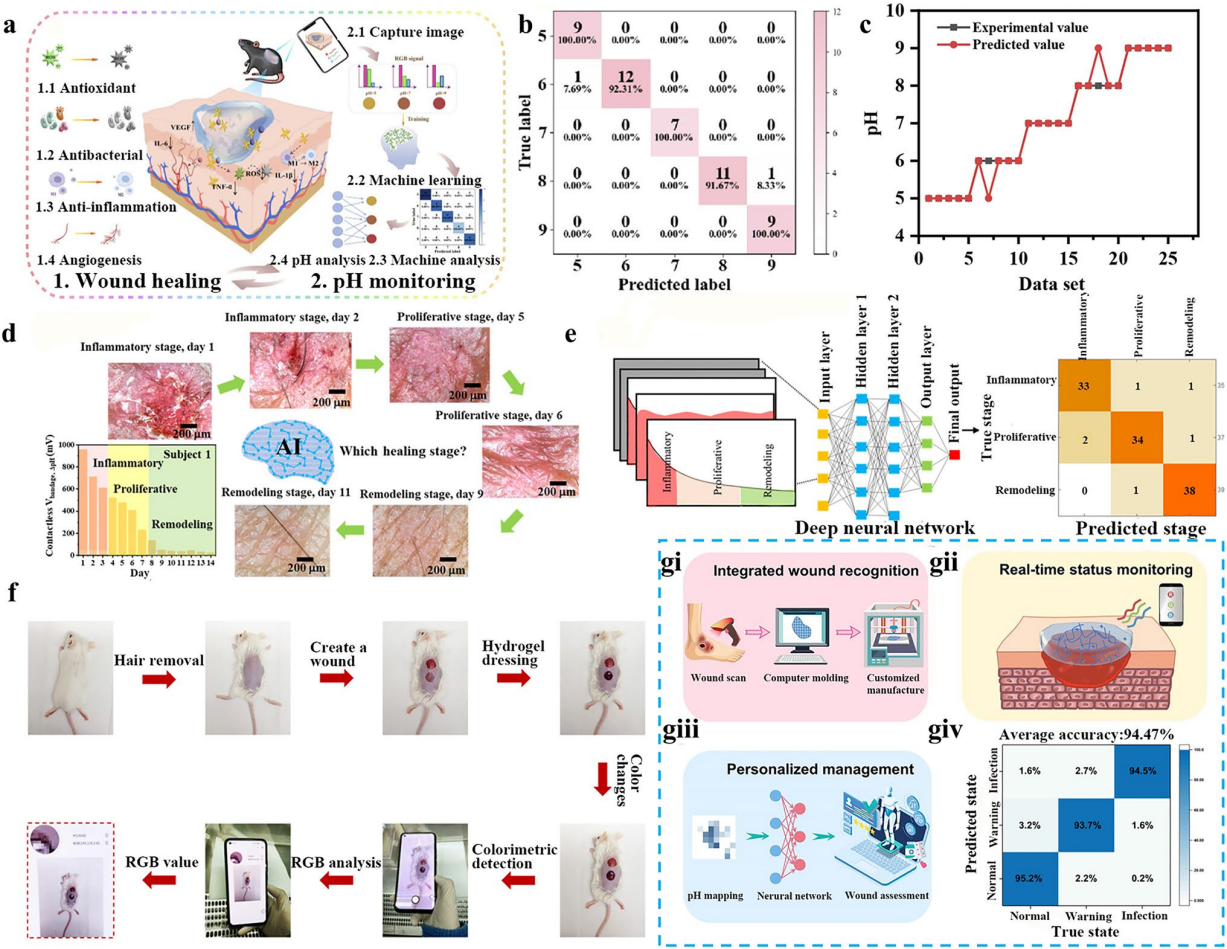
AI-assisted wounds monitoring. **a** Potential application mechanism in diabetic wound healing. **b** Confusion matrix of KNN prediction for pH 5–9. **c** Comparison of predicted values from the KNN model versus experimental values for pH. Reproduced with permission from Ref. [30]. Copyright 2025, Elsevier. **d** Output predicted healing stages from the contactless measurement of this intelligent pair collected from an inflamed skin subject. **e** A confusion matrix to classify inflammatory, proliferative, and remodeling stages computed by the deep ANN algorithm. Reproduced with permission from Ref. [27]. Copyright 2022, American Chemical Society. **f** Experimental process of wound healing in vivo under intelligent wound monitoring. A schematic diagram of intelligent wound monitoring with multifunctional hydrogel as wound dressing, including **gi** wound recognition, **gii** real-time status monitoring and **giii** personalized wound management. **giv** Confusion matrix for the wound pH mapping shows the accuracy of the CNN. Reproduced with permission from Ref. [26]. Copyright 2022, Elsevier

The k-nearest neighbors (KNN) technique is characterized by its simplicity and convenience. It does not necessitate complex training procedures. With flexible data requirements, it can accommodate various amounts of data. Its core mechanism hinges on the measurement of the distance among data points. Specifically, it conducts predictions by identifying several neighboring data points that exhibit the highest degree of similarity to the target data point. Moreover, it is suitable for both quantitative and qualitative data, demonstrating its adaptability to diverse data types. Equipped with real-time and dynamic monitoring capabilities, it can immediately respond to new data, adapt to dynamic changes in wounds, timely warn of abnormal conditions, and is appropriate for primary healthcare and home care. One crucial factor that can reveal the condition of the wound is the pH value. Chronic wounds have a relatively alkaline pH, which progressively changes to neutral as the wound heals and then turns acidic [46, 118]. By observing the pH level at the wound site, Deng et al. [30] were able to promptly identify the wound site’s degradation. The color of phenol red, which is sensitive to pH, changes from yellow to orange and red when the pH rises between 5 and 9. A mobile device was utilized to capture the color change of the hydrogel with metal nanoparticles. Subsequently, the RGB values were measured. Then, the functional relationship between the RGB values and the pH of the wound was determined (Fig. 8a). Finally, the KNN model was employed to evaluate the accuracy of the hydrogel RGB signals at different pH values. The prediction accuracy can reach 96% (Fig. 8b). To achieve the goal of exact wound monitoring, it is suggested that machine learning can precisely monitor and analyze changes in pH values at the wound site. Additionally, ongoing training and data collecting can increase prediction accuracy (Fig. 8c).

Artificial neural network (ANN) architectures, emulating biological neuronal interconnectivity through adaptive weighted node systems, demonstrate particular efficacy in processing heterogeneous biomedical data streams. In comparison to machine learning, this computational framework empowers real-time analysis of high-dimensional sensor outputs in the wound monitoring process. This is accomplished through multimodal data fusion and adaptive signal filtering. Concurrently, it enhances diagnostic accuracy through pattern recognition and optimizes cost-efficiency through edge computing deployment. In patients with psoriasis, eczema, dermatitis, and other non-communicable rashes, Kalasin et al. [27] separated the healing process into three phases: remodeling, proliferation, and inflammation. To evaluate the skin healing stages in real time, pH responsive voltage was used as a crucial sensing potential for each stage: the skin is covered with oily, white, scab-like patches during the inflammatory stage, and a sensing potential of about 985 mV was found. The conductive hydrogel dressing detected a potential of roughly 496 mV during the proliferation phase. When fibroblasts repaired inflamed skin and the sensor detected a potential of roughly 48 mV, the healing process progressed into the remodeling stage (Fig. 8d). Sensing potential can be used to differentiate and assess the wound healing status of patients with chronic inflammatory skin in this investigation. Although there was an exponential decay relationship between non-contact potential and rash size in patients with psoriasis and scleroderma, the non-contact potential of contact rash and urticaria showed a rapid linear decay with the reduction of rash, significantly decreasing, following treatment with corticosteroids. To accomplish intelligent real-time wound monitoring, the researchers combined this conductive hydrogel sensor with the deep learning ANN algorithm. The deep learning model illustrated the cure phase diagram of skin disorders that were fast cured, slowly cured, and uncured. It was built using the input data of non-contact potential captured by the MCU unit. The conductive hydrogel dressing with cuprous ions and PANI distinguished various wound types, forecasted the stage of wound healing, and measured the relationship between non-contact potential and time. The non-contact measurement accuracy of various skin disease wounds was 94.6%, according to the associated confusion matrix for healing stage recognition (Fig. 8e). By monitoring patients’ wounds state before clinical evaluation, avoiding inadequate clinical preparation, and seeking appropriate intervention when skin diseases cannot be treated with dermatology drugs, the wearable conductive hydrogel dressing inspired by this allowed medical staff to have a more thorough judgment on wound healing before clinical intervention, which was conducive to the development of clinical treatment.

Convolutional neural network (CNN) [115, 119], as a specialized ANN architecture, demonstrates exceptional proficiency in processing wound image data with a grid structure through automated feature learning and extraction. This capability enables high-precision classification of wound images and predictive analysis of healing progression. The integration of CNN technology with pH-responsive hydrogel monitoring systems, which correlate chromatic transitions (RGB variations) with biochemical changes during wound repair, presents significant clinical potential for intelligent wound management platforms. Using the solvent displacement approach, Wang et al. [26] added litmus reagent to the multifunctional hydrogel network before using a smartphone to read the hydrogel’s RGB value (Fig. 8f). Only the blue signal of the multifunctional hydrogel rose as the pH value rose during the RGB signal monitoring procedure. By fitting the link between the blue signal’s response intensity and the pH value, the study team standardized the colorimetric signal. They also found a linear relationship between the pH value and the B value and the accompanying equation that was used to determine the actual wound pH value. Using a smartphone to extract the RGB value of the corresponding position, the colorimetric hydrogel dressing was manually divided into a 4 × 3 array distribution, and a pH sensing map was calculated and drawn based on the standard curve to estimate the specific state of the wound (healing or infection). CNN technology was used to develop a machine learning model for personalized wound management, which can achieve real-time wound monitoring and evaluation to avoid the burden that frequent medical visits and continuous monitoring place on patients and healthcare systems. A pH of 4 to 6 in the wound bed was deemed typical for this investigation. A pH reading between 6 and 7.5 showed a warning status and suggested an infection risk. It was determined that an infection state was present when the pH value was between 7.5 and 9 [120, 121]. The CNN model in the personalized wound management system can extract and learn predefined features from the input data signal to output corresponding recognition results through its convolutional layer. Following CNN machine learning algorithm training, the system intelligently evaluated and predicted the wound status (normal, warning, and infection) through the trained prediction model based on the pH colorimetric signal collected by the multi-functional hydrogel dressing (Fig. 8gi-giii). This procedure was carried out using a data set consisting of 2400 pH sensing images, all of which were randomly selected as training and testing samples in an 80:20 ratio. More significantly, tailored wound management was effectively demonstrated by using the CNN deep learning algorithm for signal analysis, which achieved an accuracy rate of 94.47% in assessing wound infection and healing (Fig. 8giv). Consequently, for effective wound treatment and healthcare applications, this deep learning-based individualized wound management gave patients and healthcare practitioners more straightforward information about wound healing.

In conclusion, creating an intelligent conductive hydrogel sensor network for wound monitoring requires the integration of AI. Beyond increasing the precision and effectiveness of wound assessment, the combination of AI and intelligent sensors helps create data-driven, individualized healthcare plans. Medical care practices have transformed with the introduction of sophisticated real-time evaluation and individualized treatment technologies. It is anticipated that this novel approach would enhance wound care and patient outcomes, representing a significant advancement in the medical industry. However, currently AI mainly develops models based on a single or few physiological signals, for example, temperature, pH, pressure, etc., ignoring other physiological signals in the wound microenvironment that may increase the risk of wound misjudgment. Existing AI models focus on healing assessment and infection recognition, lacking coverage of complex scenarios. At present, wound monitoring systems are mainly based on traditional machine learning (KNN) and basic deep learning (CNN), with less application of cross modal fusion algorithms. The future conductive hydrogel wound monitoring research will achieve the upgrade to “full dimension intelligent management” by combining AI to build a closed-loop system of “signal acquisition-intelligent analysis-precise intervention”.

## Refractory Wound Applications

Refractory wounds are those that are unable to be treated within the typical healing period by standard treatment techniques. The wound-healing process is often delayed due to a multitude of factors. These encompass mixed infections at the wound location, difficulties in treating underlying diseases, and other related issues. Researchers have shown that conductive hydrogel dressings have a wide range of potential and are efficient in healing refractory wounds. The common forms of chronic wounds, for instance, pressure ulcers, diabetic ulcers, and articular wounds, will be covered in this part. Conductive hydrogel dressings will also be investigated to facilitate the healing and monitoring of chronic wounds.

### Pressure Ulcers

Pressure ulcers typically manifest in areas where bones are prominent, such as the sacrum, buttocks, and heels [122]. Prolonged pressure, friction, or shear forces can create pressure ulcers by damaging the blood flow to the affected area and causing tissue starvation. Patients experiencing unconsciousness, paralysis, mobility impairments, or those who are on prolonged bed rest are at a higher risk of developing this type of pressure ulcer. These patients are unable to fully release the pressure area, and prolonged pressure exposure to a particular body part might disrupt local blood circulation, resulting in some biochemical changes that can cause ulcers and tissue damage [123]. The initial stage of pressure ulcers is congestion and redness, which primarily manifests as localized skin swelling, discomfort, and numbness. These stages are separated based on the severity of the wound. In the second stage, known as the inflammatory infiltration stage, the skin will turn purple and the affected area may feel uncomfortable and hard. The third stage is referred to as the superficial ulcer stage. In this stage, the subcutaneous tissue becomes exposed, and an ulcer is formed in the affected area. The fourth stage is known as the necrotic ulcer stage, patients may exhibit signs of necrosis in the afflicted location, which could potentially result in sepsis [124]. Malnutrition, prolonged bed rest, and even paraplegia are experienced by the patients themselves. Some people who suffer from nerve loss also face challenging financial circumstances. Refractory wound healing is caused by both social and physiological reasons. Therefore, at this historic juncture, there is a need for conductive hydrogel dressings that combine pressure ulcer therapy and monitoring.

**Fig. 9:**
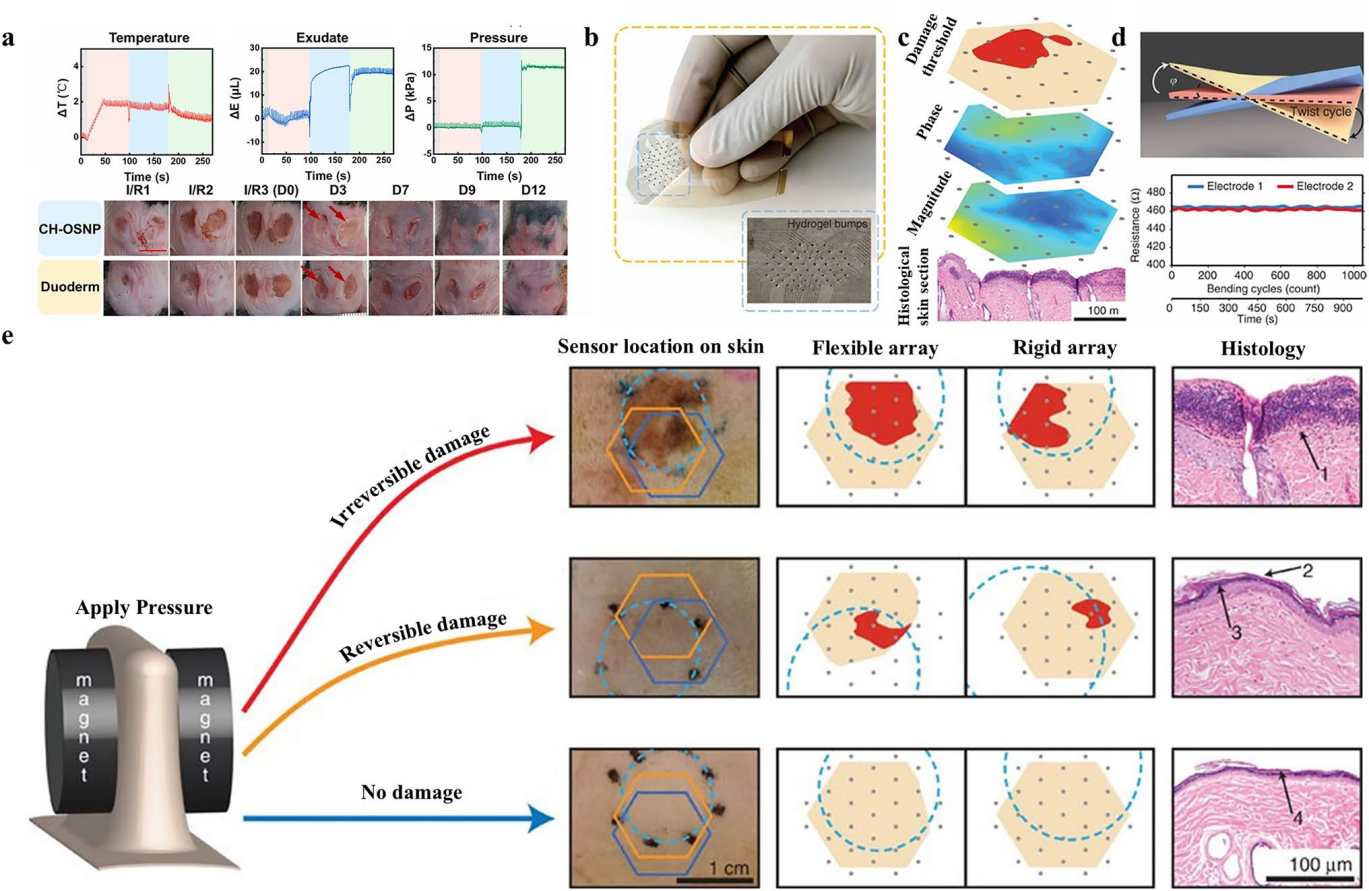
Conductive hydrogel dressings for pressure ulcers. **a** Continuous real-time monitoring and differentiation of temperature (T), exudate (E), and pressure (P) metrics. Photographs of PIs formed and processed with hydrogel skin sensor dressings at different times. Reproduced with permission from Ref. [18]. Copyright 2022, Elsevier. **b** Schematic of the hydrogel bumps printed on the prefabricated arrays made by the device. **c** Arrays were placed on the in vivo wounds and the electrical impedance of each pair of neighboring electrodes was collected. The mapping of impedance amplitude, phase angle, and damage threshold (shown here in red) was constructed based on the location of each measurement pair. **d** Hydrogel array impedance sensing was tested for mechanical stability, and was shown to be highly stable. **e** Early detection of pressure-induced tissue damage. Representative wound images with sensor locations indicated, damage parameter maps obtained from those wounds using flexible and rigid sensor arrays, and histology results are shown for each scenario. Dashed light blue circles indicate where the pressure was applied to the skin, orange and dark blue hexagons indicate the location of the flexible and rigid arrays on the wound, respectively, and gray dots represent the electrodes. Scale bars, 1 cm (for wound photos) and 100 μm (for histology images). Reproduced with permission from Ref. [104]. Copyright 2015, Nature Publishing Group

Since prolonged, localized, continuous pressure is a major contributor to pressure ulcers, conductive hydrogel dressings currently primarily prevent pressure ulcers by monitoring the pressure signal at the lesion. Monitoring pressure is critical since pressure release-induced reperfusion injury can lead to local congestion and edema, both of which are key contributors to pressure ulcer development [125]. At several sites where pressure ulcers have already developed or are likely to develop, patients wear wireless sensors. Through the use of an A/D converter, these sensors will track the pressure of the wounds over time and transform the data into digital signals that will be wirelessly sent to the healthcare system’s base station. When the sensor detects high pressure at the wound site, which is detrimental to the tissue, it immediately alerts the medical staff to reposition the patient (Fig. 9b-e). This timely action is crucial to prevent pressure ulcers. By encasing the NFY network in a conductive hydrogel sensor, Qiu et al. [16] created a 3D hybrid scaffold demonstrating dual functionality: antimicrobial action to accelerate infected wound healing and precise modulation of cellular proliferation and spatial reorganization. By incorporating NFY and ions, this study endowed the hydrogel with exceptional conductivity, enabling it to serve as a flexible strain sensor that accurately monitored wound pressure while accelerating pressure ulcer healing, thus achieving the goal of integrated treatment and monitoring. It is challenging to repair the nerve damage elicited by pressure ulcers, thus it is helpful to focus on the nerve signals at the wound site of patients to prevent both structural and functional harm to the damaged nerves. A double crosslinked and double conductive hydrogel system composed of GO and PEDOT was created by Lei et al. [56]. This technology enabled continuous nerve signal monitoring for wound condition assessment since the peripheral nerve fibers were seamlessly laminated. Place the hydrogel scaffold membrane electrode assembly pad on the rat sciatic nerve after attaching it to the specially made flexible flat circuit board and covering it with PDMS. In the frequency range of 100 Hz to 10 kHz, the hydrogel MEA exhibited a measured impedance response of roughly 1 to 2 kω, which was consistent with the values seen in vitro. Bipolar electrodes were used to record induced composite neural action potentials, which were then analyzed using the fast Fourier transform. The frequency power accumulation showed that all electrode distance settings (D1, D2, and D3) were capable of accurately measuring the compressive force events of the stimulated condition in comparison to the unstimulated control condition. According to these findings, hydrogel scaffold MEAs can establish conformal contact with nerve tissue to enable site-specific signal recording. The synergistic effect of electrical stimulation and cell transplantation based on spatiotemporal control can also aid in neural function recovery. This conductive hydrogel dressing was used at the pressure sore site to record the changes in nerve signals at the wound site in real time through the stimulated compression force event, providing valuable information for the monitoring and management of nerve function at the pressure sore site. Building on the foundation of single-factor monitoring, Liu et al. [18] developed a conductive hydrogel dressing that enables continuous, remote, and real-time monitoring of temperature, pressure, exudate, and other indicators while treating pressure ulcers (Fig. 9a).

As demonstrated above, the conductive hydrogel dressing has advanced significantly in the monitoring and management of pressure ulcers. The conductive hydrogel dressing currently not only enables single monitoring of pressure and nerve damage in pressure ulcers, but it can also accomplish multi-factor monitoring and precise differentiation. The combination of pertinent sensors for promoting ulcer healing also enhances the function of such sensors, which is advantageous for patients with pressure ulcers requiring home care and rehabilitation, particularly those suffering from dementia or sensory loss. Deep learning is now hardly ever integrated into pressure ulcer monitoring systems, which means that post-injury evaluation is the primary basis for pressure ulcer wound monitoring. To improve diagnostic accuracy, reduce decision-making time, increase monitoring dimensions, and advance intelligent wound care, researchers can eventually integrate AI with monitoring systems through feature learning, along with data images, to accomplish the goal of “quantitative prediction” of pressure ulcers in high-risk populations as early as possible.

### Diabetic Ulcers

Diabetes, a metabolic disorder characterized by hyperglycemia, affects hundreds of millions worldwide, posing significant challenges and economic burdens globally [126]. Diabetic ulcers are one of the diabetes complications, which can make people weak and lead to an increase in the incidence rate of other diseases [127]. Therefore, it is an urgent problem to monitor and treat chronic diabetic ulcers. Elevated local blood glucose levels in diabetic ulcers induce cellular membrane sclerosis, impeding blood flow through critical microvessels in the wound bed. This mechanism ultimately leads to impaired healing of diabetic ulcers [24].

**Fig. 10:**
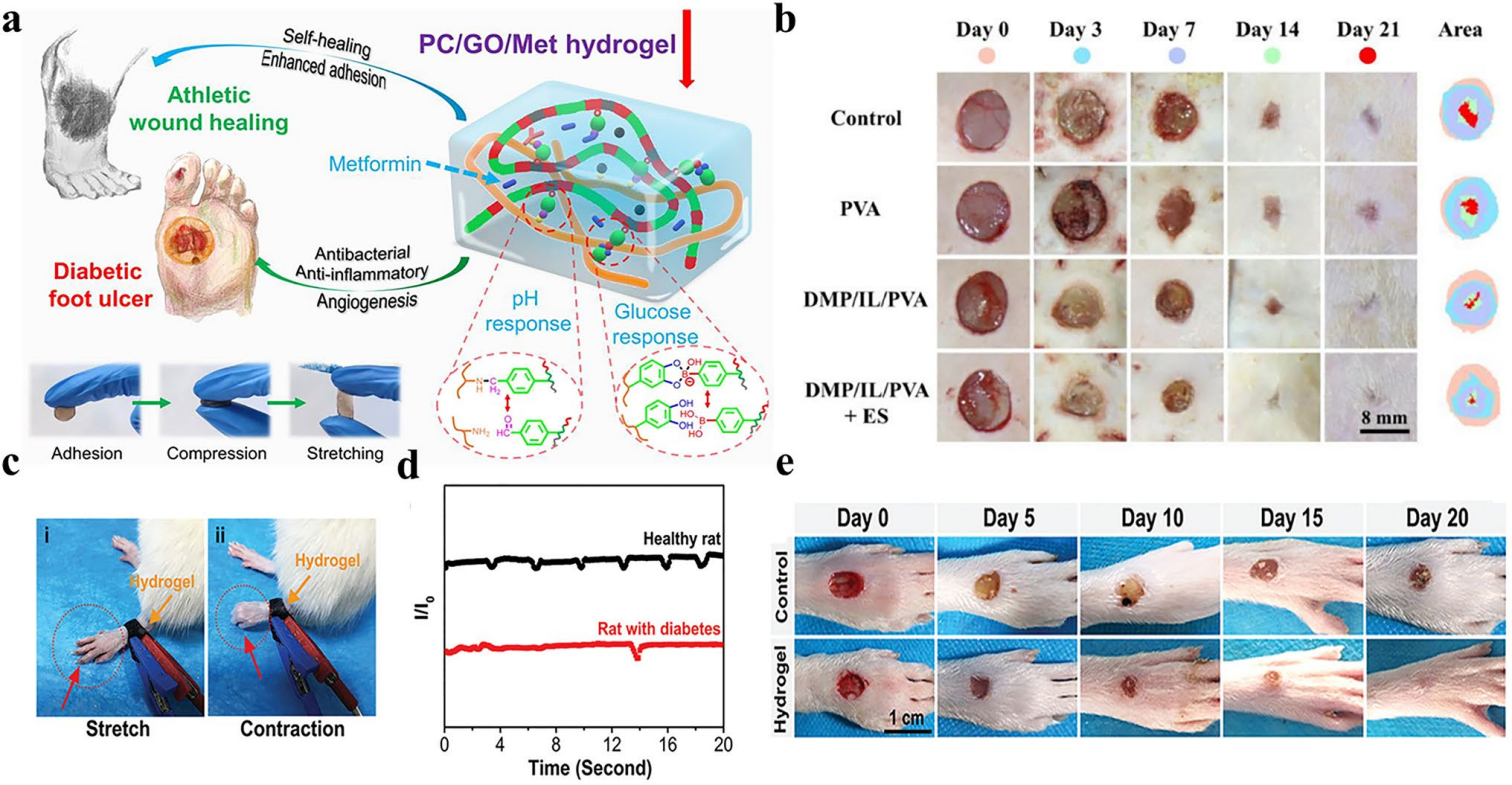
Conductive hydrogel dressings for diabetic ulcers. **a** Schematic representation of the structure, pH and glucose response mechanism of PC hydrogel and its application in diabetic foot ulcers and sports wound healing. Reproduced with permission from Ref. [127]. Copyright 2022, American Chemical Society. **b** Hydrogel dressing for treatment of diabetic wounds as well as wound size monitoring. Reproduced with permission from Ref. [80]. Copyright 2023, Elsevier. **c**, **d** Due to nerve damage, diabetic rats are slower to respond to environmental stimuli compared to normal rats. The designed conductive hydrogel dressing can reliably reflect the lower motor reflex frequency of diabetic rats during continuous thermal stimulation. **e** Representative photographs of diabetic feet after treatment with PBS and conductive hydrogel dressing, the conductive hydrogel dressing group significantly promotes wound healing. Reproduced with permission from Ref. [64]. Copyright 2019, Wiley–VCH

Monitoring the glucose concentration within the wound holds significant importance in the treatment of diabetic ulcers. Since the wound management strategy can be promptly adjusted according to the local blood glucose level (Fig. 10a) [127]. An extremely transparent conductive hydrogel patch was created by Liu et al. [70]. When this conductive hydrogel dressing came into contact with glucose at several concentrations (20, 50, 100, and 200 mM), the δR/R_0_ exhibited a regular decreasing trend with a good linear connection as the glucose level at the wound can mirror the blood glucose level. Real-time monitoring is possible by correctly detecting and wirelessly transmitting the changes in the wound’s glucose level to a mobile device. Owing to the outstanding transparency of the conductive hydrogel, the research team successfully carried out the monitoring of wound healing from both macroscopic and microscopic perspectives. By speeding up hemostasis, enhancing intercellular communication, preventing wound infection, encouraging collagen deposition, and stimulating angiogenesis, this conductive hydrogel dressing not only resolved the “black box” state of the healing process and enabled indirect blood glucose monitoring by measuring the glucose level at the wound, but it also successfully accelerated the healing of diabetic ulcers.

The oxidative microenvironment hinders diabetic skin lesions from healing besides altering the glucose level at the site [128]. In the presence of glucose, the endogenous oxidoreductase glucose oxidase (Gox) can initiate a cascade process that generates ROS. ROS affects how pain is perceived, and the pain signal produced at the diabetic ulcer aids in real-time wound healing status monitoring (Fig. 10b) [80]. When nanoparticles are added to the hydrogel system, they can identify the mild pain signal that results from the diabetic wound cascade response and output the change in this signal as a resistance waveform change. As a result, tracking the resistance waveform change can help determine how ROS is changing at the wound, explain how the pain signal is changing there, and indirectly represent the wound’s current state (Fig. 10c-e) [64, 129]. To monitor the force and size of diabetic ulcer wounds and to achieve real-time monitoring of diabetic ulcer wounds through relative resistance change, some researchers have also created hydrogel array sensors. This conductive hydrogel dressing with ion conductive material can precisely monitor the wound while promoting wound healing, accelerating angiogenesis, and stimulating cell migration and proliferation [101]. The dressing demonstrated both rapid, precise real-time responsiveness to wound changes and excellent antibacterial properties, making it highly beneficial for treating diabetic ulcer wounds. Moreover, the resistance change of a conductive hydrogel dressing with CNT/PEDOT: PSS dual-conductive network can be utilized to monitor wound pH and temperature [47].

In addition to accurately judging and evaluating the healing status of diabetic ulcers, it can also promote wound healing with various functional groups of conductive hydrogel dressing addition, achieving the integration of monitoring and promoting healing. It does this by monitoring various diabetic wound signals, glucose levels, pain signals, pH, and temperature, and by producing real-time signals. The current body of researches is crucial to advancing the use of conductive hydrogel dressings in diabetic ulcer wounds. Hydrogel wound dressings, however, rarely accomplish multidimensional parameter monitoring in diabetic ulcer monitoring research at this time. Instead, they primarily focus on one or a small number of parameters (temperature, pH, glucose level, etc.) and do not provide thorough monitoring of the inflammatory factors concentration, blood oxygen saturation, tissue fluid metabolites, and other important dimensions in the wound microenvironment. Future studies should shift from “single point monitoring” to “systematic monitoring” and focus more on the multifaceted improvement of conductive hydrogel dressings in diabetic ulcer monitoring.

### Articular Wounds

**Fig. 11:**
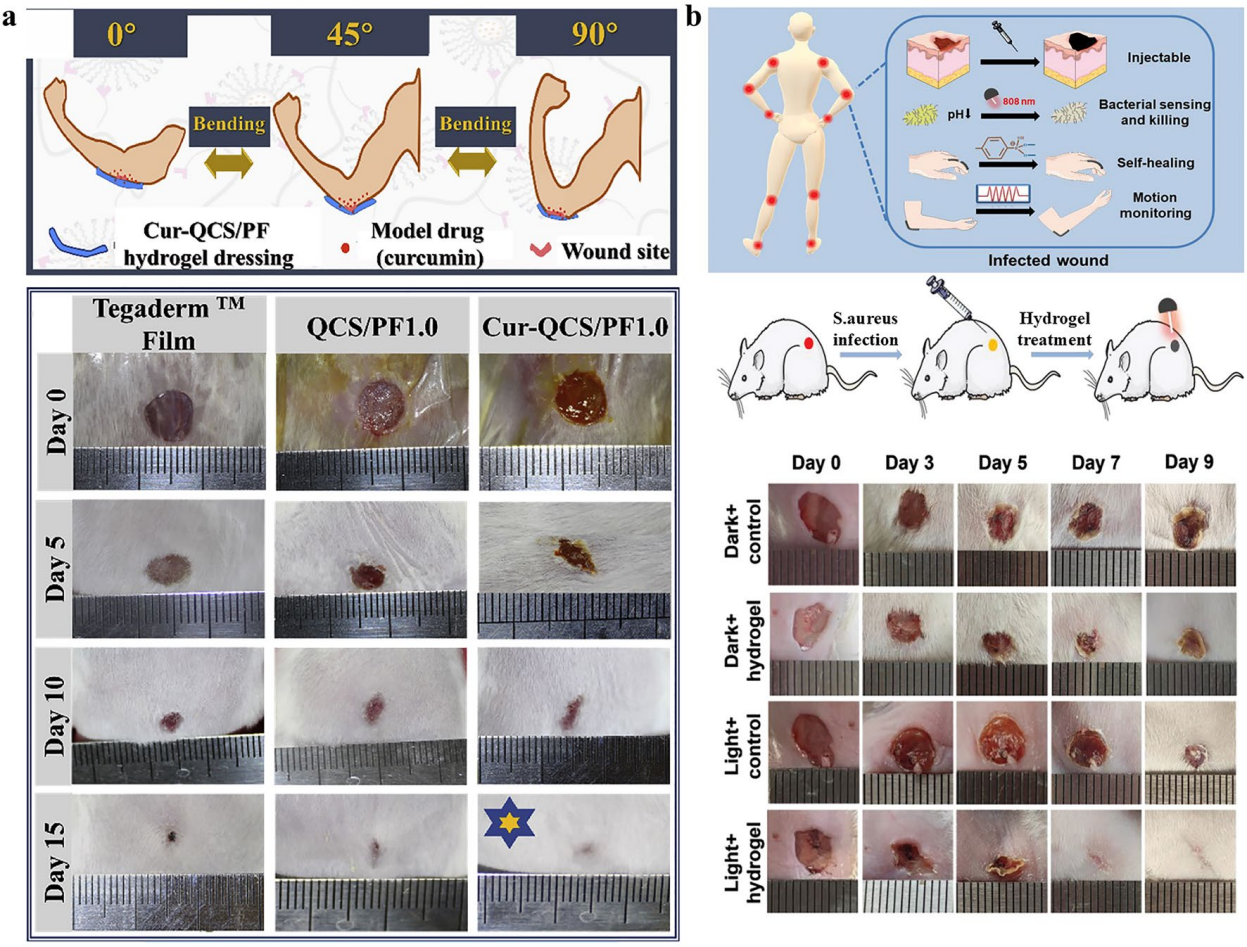
Conductive hydrogel dressings for articular wounds. **a** Conductive hydrogel dressings containing curcumin can not only monitor wounds by joint motion status but also release curcumin through pH changes at the wound site for therapeutic purposes. Reproduced with permission from Ref. [132]. Copyright 2018, Elsevier. **b** Hydrogel dressing realizes real-time monitoring of wounds in body joints and prevents retearing of wounds in human joints, and realizes in situ bacterial sensing and killing functions by changing electrical signals and photothermal Therapy. Reproduced with permission from Ref. [133]. Copyright 2024, Wiley–VCH

Due to the high degree of mobility in the fingers, elbows, and knees joints, wounds in these areas often experience a sluggish healing process and pose greater difficulties in treatment [130]. Higher mechanical qualities of dressings are also necessary for the unique nature of joints. It requires intrinsic hyperelasticity and tissue-conformal adhesion to maintain joint mobility and prevent mechanical detachment during dynamic movement [131]. Articular wounds can be sensed and autonomously repaired using conductive hydrogel dressings with motion monitoring features. When applied to articular wounds, it prevents repeated movements from causing dressings to detach or lose tight adherence to the wound site, increasing the risk of cracking or even retearing and secondary infection, which would prolong the healing process and create more discomfort [132]. By speeding up cell migration, proliferation, and regeneration, the conductive groups in the hydrogel can also aid in wound healing and provide efficient wound care (Fig. 11a).

One popular technique for impedance analysis in biosensors is electrochemical impedance spectroscopy (EIS). To obtain the conductivity of the entire dressing along the fiber axis, Ma et al. [19] added silver nanoparticles to the spiral fiber composite coating. Apart from outstanding conductivity, the complete conductive hydrogel dressing had excellent extensibility, allowing it to adjust to a variety of big joint movements. Motion monitoring was made possible by the constant current, which caused the voltage variation between the dressing’s two ends to reflect resistance changes. Three distinct movement kinds were observed by the research team: twisting, folding, and stretching. Compared to the other two movements, the amplitude of the joint extension movement was greater, and signals of varying heights reflected amplitudes. The associated electrical signal varied more significantly with increasing elbow bending angle. The signal peak resembled that of joint bending and relaxation when the wrist was raised to fold the bandage, but the trough was more erratic. When the wrist is moved laterally, an electrochemical workstation is capable of recording the stretching and compression occurring on one side of the dressing. The twisting motion’s reduced amplitude meant that the electrical signal shift was not as noticeable as it was for the first two motions. All three distinct movements can be distinguished based on either variations in resistance or variations in electrical impulses. This capability aids physicians in evaluating patients’ motions and accelerating the restoration of their motor skills.

Due to the irregular shapes of articular wounds and the frequent movements of joints, ordinary dressings struggle to adapt to the contours of these wounds. This situation elevates the risk of infection at the joint wound sites. By adding graphene to the hydrogel network and using conductive hydrogel sensors, Shan et al. [133] achieved the goal of combining sensing and sterilization. By combining the detection of bacteria and sterilization into a single system, the diagnostic antibacterial hydrogel can improve sterilization efficiency and overcome the drawbacks of separate, expensive, and complicated diagnosis and sterilization procedures in conventional methods. The hydrogel dressing was injectable and could precisely conform to the wound shape. The development, reproduction, and metabolism of bacteria in an infected wound resulted in the creation of acidic chemicals, which progressively lowered the surrounding environment pH. When the pH of the hydrogel in contact with the bacterial site dropped below the pKa (approximately 8.5) of the phenyl borate group, the borate ester bond was cleaved, and the hydrogel network underwent partial dissociation. Sol–gel transformation made it possible to detect germs, drastically lowered resistance, and sped up the flow of electrons in graphene (Fig. 11b). Through alterations in electrical impulses, this kind of sensor may identify bacterial infections. It can also make decisions in real time and track how well articular wounds are healing. In situ photothermal sterilization can be used if bacterial infections were found while the lesion was healing. Excellent clinical application prospects had been demonstrated by the system that integrated the diagnosis and treatment of bacterial infections. Besides, appropriate sterilization after infection, which inhibits bacterial growth and prevents the emergence of drug-resistant bacteria, along with monitoring bacterial infections in wounds, helps avoid poor articular wound healing. Similar to this, when applied to a stretchable part of the human body, this conductive hydrogel dressing may track joint movement as an articular wound dressing, preventing secondary fractures. The conductive hydrogel sensor in this study can detect different bending angles of the joint in a timely and evident manner since the hydrogel reacted quickly to the stimulation caused by the joint bending movement, the response signal accurately corresponded to each movement, and the sensing response increased proportionally with the bending angle. This study presented a novel multipurpose hydrogel dressing that can detect and cure bacterial infections, encourage the healing of articular wounds, and sensitively measure joint movement strength to prevent the wound from ripping again. This demonstrates how individualized wound care can be achieved by combining electronics and therapy. Clinical application prospects for intelligent healthcare, wearable electronics, and customized wound care are excellent.

In summary, owing to their distinct mobility features, articular wounds present numerous treatment issues. Innovative approaches to treating articular wounds have been made possible by the conductive hydrogel dressing, which serves as both a therapeutic and monitoring tool. It enables concurrent monitoring of dynamic biomechanical signals at articulating joints and infection-induced pH fluctuations at wound sites, while maintaining conformal adaptation to irregular wound morphologies. This provides compelling evidence for prompt treatment plan adjustments. The accuracy of wound state diagnosis is limited, nonetheless, by the absence of composite and thorough important variables monitoring comprising blood oxygen saturation and wound site temperature with the present monitoring parameters for articular wounds. Furthermore, data fusion and AI algorithms are lacking for additional backend data processing and prediction. To address the shortcomings in pertinent areas and enhance articular wounds care and monitoring, future researchers should carry out comprehensive studies.

In the final analysis, the chronic presence of pressure factors at the wound site causes pressure ulcers to hinder wound healing, increasing the risk of infection and making nursing care challenging. In addition to the direct monitoring of wound pressure, real-time monitoring of wound temperature and exudate also holds significant clinical value. This is attributed to the potential infection risk. Diabetic ulcers are caused by the underlying condition of diabetes, which persists and causes high blood sugar at the lesion, making it challenging to heal. Therefore, to understand the inflammation and healing status of a diabetic ulcer, it is possible to monitor the glucose level at the site along with certain biomarkers. Due to the high blood circulation in the joint region and the frequent joint activities, newly formed tissues in the joint area are susceptible to secondary injury caused by stretching. Monitoring the pressure and bacteria at the joint wound site is crucial since the possible infection danger makes therapy more complicated. In treatment, the conductive hydrogel dressing integrates multiple therapeutic mechanisms to enhance efficacy. In monitoring, it overcomes the limitations of conventional wound monitoring, such as the need for complex external devices—enabling in situ, real-time, and non-invasive tracking that improves patient outcomes and treatment experiences. However, when it comes to the current monitoring methods of conductive hydrogel dressings applied to refractory wounds, several notable challenges and limitations persist. It is challenging to appropriately manage complex wounds (such as multiple infections and rare wounds). For instance, the data processing lacks integration with AI algorithms, the monitoring parameters are rather basic, and important aspects are not monitored. In the future, as materials science and biomedical engineering continue to advance, after improving the relevant issues, it is anticipated that conductive hydrogel dressings will be extensively utilized and further optimized in clinical settings. This development is expected to bring positive outcomes for a larger number of patients suffering from refractory wounds.

## Conclusions and Outlook

This study provides an overview of the latest developments in conductive hydrogel dressings for individualized chronic refractory wound monitoring and care. Apart from successfully accelerating wound healing, the organic fusion of conductive hydrogel dressings and intelligent sensors allows for ongoing, real-time wound monitoring. Based on the monitoring results, these conductive hydrogel dressings equipped with wound-monitoring capabilities can support treatment to effectively control the wound environment. Although these smart sensor dressings possess considerable potential, numerous opportunities and challenges still exist in real-world clinical environments.

First of all, the stability of the sensor must be increased and the safety of the conductive hydrogel dressing must be guaranteed. Conductive hydrogel dressings for human wounds should prioritize conductive materials with high biosafety. To enhance their biocompatibility and dispersion while reducing their potential cytotoxicity, the surfaces of conductive materials constituted by metal and carbon require modification. Additionally, the stability of conductive hydrogel sensors will serve as a strong assurance for dependable wound monitoring over the long run. Although the majority of conductive hydrogels exhibit poor water-retention capabilities, this problem can be addressed by incorporating additional components. Further, appropriate materials can be added to irregular areas like joints to guarantee a precise fit between wound sections and conductive hydrogel dressings as well as the stability of signal detection. As these challenges are gradually surmounted, the wound-monitoring sensors grounded in conductive hydrogels will be empowered to operate effectively across a broader spectrum of application scenarios. Moreover, they will enjoy an extended lifespan, enabling more prolonged and reliable performance in the wound care field.

Second, incorporate signals from multidimensional monitoring. When conductive hydrogel dressings utilize a single pH-sensitive substance to track alterations in the wound microenvironment, the accuracy of monitoring can be compromised by the interference of wound exudates in measuring color changes. The assessment of therapy success will be insufficient if the association between multidimensional indicators for complex wounds cannot be thoroughly integrated and examined. Therefore, the capacity to monitor the characteristic parameters of the wound microenvironment hinges on the achievement of signal fusion among the conductive hydrogel’s data layer, feature layer, and decision layer. To acquire more extensive and comprehensive wound information, the multidimensional monitoring signal fusion of the conductive hydrogel dressing integrates and analyzes various types of monitoring signals. This not only substantially enhances the monitoring accuracy but also improves the reliability of the monitoring system. It is an indispensable technology for ensuring the precision and comprehensiveness of wound monitoring.

Third, increase the number of wound types that can be monitored using conductive hydrogel dressings. At present, the application of conductive hydrogel dressing in refractory wounds monitoring has the limitation of narrow coverage of wound types, mainly focusing on a few diseases such as pressure ulcers, diabetic ulcers and articular wounds, while there is little research on the monitoring of complex types, for instance, burns, ischemic ulcers, radiation damage, tumor-related wounds, etc. In the future, the monitoring scope can be extended from “single disease” to “all spectrum refractory wounds” by creating pathology-specific sensors and building data models for rare wound illnesses. This will offer dependable solutions for individualized monitoring and treatment of complex wounds.

Fourth, investigate more sophisticated AI algorithms to examine intricate wound characteristics in several dimensions. The application of hydrogel dressings in combination with AI technologies for real-time wound monitoring is a relatively nascent field. The combination of data processing and complex algorithms relies on the cloud, resulting in poor real-time wound monitoring, and clinical research often focuses on a single wound type, with scarce monitoring data and insufficient algorithm generalization ability for refractory wounds including burns and ischemic ulcers. As technological advancements persist, more complex sensor networks will be developed in the future to track an increasingly diverse and intricate array of wound characteristics. Moreover, it may become feasible to uncover latent correlations between parameters that are undetectable by traditional methods. Individual multidimensional wound parameters can be harnessed to provide personalized healing assessments and predictions. AI systems can rapidly analyze the collected monitoring data and offer real-time decision-making support. Through continuous learning and optimization, AI algorithms can enhance their capabilities in monitoring wound characteristics, improving prediction accuracy, and tailoring treatment plans to complex wound scenarios and evolving clinical requirements. To advance the field of wound monitoring and ensure the robustness and generalizability of AI models, it is crucial to construct a comprehensive dataset encompassing various tissue components and to explore more sophisticated AI algorithms for clinical applications.

Last but not least, encourage the clinical use of customized wound care and monitoring systems. The market for conductive hydrogel dressings is urgently in need of commercialization due to the rise in medical demand and the quick development of related technologies. The majority of conductive hydrogel dressings with wound monitoring and treatment capabilities, however, are still in the experimental testing and academic research stages and require more investigation and development. In addition, their production costs are relatively high, which might restrict their ability to be promoted on the market. Real-time transmission of physiological data and wound images includes patient privacy and necessitates striking a compromise between performance and security. Thus, businesses and scientific research organizations should invest more in research and development, continuously enhance product performance, lower production costs, boost the competitiveness of conductive hydrogel dressings in the market, and encourage their commercialization through process optimization and increased production efficiency. The commercialization of conductive hydrogel dressings is anticipated to have a significant impact on medical care and will alter the way that human health is monitored.

In conclusion, conductive hydrogel dressings and the Internet of Things (IoT) can be combined to improve wound monitoring’s accessibility and continuity. This will allow patients to take an active role in their care and medical professionals to monitor wounds more thoroughly. As modern technology advances, we think that AI-assisted intelligent dressings have opened the door to more effective treatment plans for chronic refractory wounds, offering the therapeutic community a bright future.

## Data Availability

No data was used for the research described in the article.
